# The role of biomarkers and dosimetry parameters in overall and progression free survival prediction for patients treated with personalized ^90^Y glass microspheres SIRT: a preliminary machine learning study

**DOI:** 10.1007/s00259-024-06805-8

**Published:** 2024-07-09

**Authors:** Zahra Mansouri, Yazdan Salimi, Ghasem Hajianfar, Nicola Bianchetto Wolf, Luisa Knappe, Genti Xhepa, Adrien Gleyzolle, Alexis Ricoeur, Valentina Garibotto, Ismini Mainta, Habib Zaidi

**Affiliations:** 1 Division of Nuclear Medicine and Molecular Imaging, Diagnostic Department, Geneva University Hospital, CH-1211 Geneva, Switzerland; 2 Service of Radiology, Geneva University Hospital, CH-1211 Geneva, Switzerland; 3 Centre for Biomedical Imaging (CIBM), Geneva, Switzerland; 4 Department of Nuclear Medicine and Molecular Imaging, University of Groningen, University Medical Center Groningen, Groningen, Netherlands; 5 Department of Nuclear Medicine, University of Southern Denmark, Odense, Denmark; 6 University Research and Innovation Center, Óbuda University, Budapest, Hungary

**Keywords:** ^90^Y Radioembolization, SIRT, Overall survival, Progression free survival, Biomarkers, Machine learning

## Abstract

**Background:**

Overall Survival (OS) and Progression-Free Survival (PFS) analyses are crucial metrics for evaluating the efficacy and impact of treatment. This study evaluated the role of clinical biomarkers and dosimetry parameters on survival outcomes of patients undergoing ^90^Y selective internal radiation therapy (SIRT).

**Materials/Methods:**

This preliminary and retrospective analysis included 17 patients with hepatocellular carcinoma (HCC) treated with ^90^Y SIRT. The patients underwent personalized treatment planning and voxel-wise dosimetry. After the procedure, the OS and PFS were evaluated. Three structures were delineated including tumoral liver (TL), normal perfused liver (NPL), and whole normal liver (WNL). 289 dose-volume constraints (DVCs) were extracted from dose-volume histograms of physical and biological effective dose (BED) maps calculated on ^99m^Tc-MAA and ^90^Y SPECT/CT images. Subsequently, the DVCs and 16 clinical biomarkers were used as features for univariate and multivariate analysis. Cox proportional hazard ratio (HR) was employed for univariate analysis. HR and the concordance index (C-Index) were calculated for each feature. Using eight different strategies, a cross-combination of various models and feature selection (FS) methods was applied for multivariate analysis. The performance of each model was assessed using an averaged C-Index on a three-fold nested cross-validation framework. The Kaplan-Meier (KM) curve was employed for univariate and machine learning (ML) model performance assessment.

**Results:**

The median OS was 11 months [95% CI: 8.5, 13.09], whereas the PFS was seven months [95% CI: 5.6, 10.98]. Univariate analysis demonstrated the presence of Ascites (HR: 9.2[1.8,47]) and the aim of SIRT (segmentectomy, lobectomy, palliative) (HR: 0.066 [0.0057, 0.78]), Aspartate aminotransferase (AST) level (HR:0.1 [0.012–0.86]), and MAA-Dose-V_205_(%)-TL (HR:8.5[1,72]) as predictors for OS. ^90^Y-derived parameters were associated with PFS but not with OS. MAA-Dose-V_205_(%)-WNL, MAA-BED-V_400_(%)-WNL with (HR:13 [1.5–120]) and ^90^Y-Dose-mean-TL, ^90^Y-D_50_-TL-Gy, ^90^Y-Dose-V_205_(%)-TL, ^90^Y-Dose- D_50_-TL-Gy, and ^90^Y-BED-V_400_(%)-TL (HR:15 [1.8–120]) were highly associated with PFS among dosimetry parameters. The highest C-index observed in multivariate analysis using ML was 0.94 ± 0.13 obtained from Variable Hunting-variable-importance (VH.VIMP) FS and Cox Proportional Hazard model predicting OS, using clinical features. However, the combination of VH. VIMP FS method with a Generalized Linear Model Network model predicting OS using Therapy strategy features outperformed the other models in terms of both C-index and stratification of KM curves (C-Index: 0.93 ± 0.14 and log-rank p-value of 0.023 for KM curve stratification).

**Conclusion:**

This preliminary study confirmed the role played by baseline clinical biomarkers and dosimetry parameters in predicting the treatment outcome, paving the way for the establishment of a dose-effect relationship. In addition, the feasibility of using ML along with these features was demonstrated as a helpful tool in the clinical management of patients, both prior to and following ^90^Y-SIRT.

**Supplementary Information:**

The online version contains supplementary material available at 10.1007/s00259-024-06805-8.

## Introduction

Liver cancer is one of the most common cancers worldwide, with a substantially increasing incident rate. Hepatocellular carcinoma (HCC), in particular, stands out as a leading contributor to cancer-related mortality [1, 2]. Selective internal radiation therapy (SIRT) through intrahepatic arterial injection of ^90^Y microspheres has been used for several decades as a treatment option [3, 4]. The safety and effectiveness of SIRT for primary and metastatic liver cancer with promising post-therapy overall survival (OS) is evident [5, 6]. The treatment has demonstrated more effective results by developing personalized dosimetry [7]. The goal of the treatment is delivering a lethal absorbed dose to the tumors while sparing the healthy liver tissue by tailoring the absorbed dose values to each specific individual [7–10].

The SIRT procedure is always simulated by similarly injected technetium-99 (^99m^Tc) macro aggregated albumin (MAA) to perform a pre-therapy scan (planar and SPECT/CT). The main objectives are to identify patients with high lung shunt and/or gastrointestinal shunt, who are contraindicated to SIRT, and recently to perform personalized treatment planning and pre-therapy dosimetry [7, 10, 11].

After therapy, the patients undergo follow-up imaging, including multiphasic contrast-enhanced CT or MRI and ^18^F-FDG PET/CT to evaluate the response to treatment. The response is reflected in changes in total lesion glycolysis measured on follow-up ^18^F-FDG PET, as well as tumor response as assessed by different versions of Response Evaluation Criteria in Solid Tumors (RECIST) [12–17]. The primary clinical objectives of follow-up imaging are the evaluation of tumor local control and early identification of non-responders to the treatment. The aim is to enhance the decision-making process. Nonetheless, the ultimate endpoint is the appraisal of overall survival (OS) [17–19].

Many institutions across the world utilize biomarkers, such as baseline tumor stage, liver function, tumor markers and performance status as the cornerstones for prognostication and survival assessment and making decisions for treatment options of an individual accordingly [20]. The OS and progression free survival (PFS) of patients undergoing ^90^Y SIRT in its different aspects, using these biomarkers has been evaluated in previous studies [21–27].

Nowadays as dosimetry for ^90^Y-SIRT patients advances, specifically with developing personalized dosimetry, absorbed dose thresholds are used for treatment planning and treatment verification. These thresholds, known as dose-volume constraints (DVCs) to the tumor and organs at risk (OAR), were largely derived from external beam radiotherapy (EBRT) practices. However, due to significant clinical variations in radiation quality, dose rate, and other factors between EBRT and SIRT, the biological responses and implications of these two treatments is different [28–30]. Consequently, and as evidence strongly support the presence of absorbed dose-effect correlation, investigations have delved into establishing an absorbed dose threshold for achieving responses in ^90^Y-SIRT [7, 31–33]. Such correlations indicate that personalized dosimetry-based treatments would improve the outcomes and increase the survival [34].

Recent studies have shown promising results regarding the correlation between tumor mean absorbed dose and OS or PFS [35, 36]. Allimant et al. conducted a comprehensive evaluation of PFS and OS in 45 patients, revealing a significant increase in both parameters with complete tumor targeting (PFS: 2.5 Vs. 7.9 months, OS: 4.5 Vs. 19.2 months) [35]. Similarly, Hermann et al. explored the relationship between tumor absorbed dose and OS in a large cohort from the SARAH trial [36]. Their results indicated that patients who received a mean absorbed dose ≥ 100 Gy with resin microspheres to the tumors exhibited an extended overall survival (14.1 vs. 6.1 months). However, current studies are limited to using statistical approaches for survival analysis considering only the association of tumor absorbed dose and survival without taking into account the corresponding absorbed dose values to normal tissues in this context.

In recent years, there has been increasing interest in applying machine learning (ML) algorithms to survival analysis tasks to assist in determining prognostic indicators [37]. Compared to conventional statistical models that struggle to capture the non-linear relationships between co-variates and output, ML models can learn the multivariate and non-linear correlations present in the training data, thus minimizing the uncertainties, and providing more robust predictions.

We therefore conducted a preliminary, yet comprehensive assessment of the contribution made by clinical biomarkers and established DVCs either for tumors and OARs in predictive modeling of the time-to-event OS and PFS outcomes for patients who underwent ^90^Y-SIRT procedures. To this end, we developed ML models that utilize the DVCs derived from ^99m^Tc-MAA and ^90^Y physical and biological effective dose (BED) maps in addition to clinical biomarkers to predict the OS and PFS.

## Materials and methods

### Patient characteristics

A retrospective study of 17 HCC patients treated with ^90^Y-glass microspheres (Therasphere™; Boston scientific group, Marlborough, Massachusetts) at Geneva University Hospital (Switzerland), between November 2021 and January 2023 was conducted. Adult patients over 18 years old with at least one tumor > 3 cm, stable liver enzymes, no contraindications to angiography, no concurrent treatment, no previous transplantation, or previous liver resection and an Eastern Cooperative Oncology Group (ECOG) performance status of 0 to 1 were included. All patients underwent personalized dosimetry and treatment planning using ^99m^Tc-MAA SPECT/CT imaging and treatment dose distributions were verified after ^90^Y treatment by Bremsstrahlung SPECT/CT imaging. Patient characteristics (including clinical biomarkers) are shown in Table 1.

**Table 1:** Patient characteristics and values of laboratory tests are reported by mean ± SD. * ECOG: Eastern Cooperative Oncology Group

Characteristics	Value
**No. of patients**	17
**Sex**	16 Males: 1 Females
**Age**	Median:72±12 [range; 39-87 yrs.]
**Etiology**	
**chronic hepatitis** **Viral** **Cirrhotic + EtOH(alcohol)** **Cirrhosis** **Noncirrhotic** **Viral and EtOH**	1 3 4 3 5 1
**Child Pugh**	
**A** **B** **Unknown**	5 2 10
**Previous treatment**	
**None** **Chemoembolization** **Chemoembolization+ immunotherapy** **Chemoembolization + sorafenib** **Previous SIRT (4 years ago)**	12 2 1 1 1
**Extrahepatic metastasis**	Non :16 Yes: 1 (lung)
**Portal vein thrombosis (PVT)**	Yes: No (5:12)
**Hepatitis**	Yes: No (4:13) (B:2, C:1, D:1)
**Ascites**	Yes: No (3:14)
**liver cirrhosis**	(No:5, yes: 12)
**Aim of SIRT**	
**Lobectomy (right: left)** **Palliative** **Segmentectomy**	12 (10 :2) 1 4
**Alpha fetoprotein (AFP) mean ± SD (µg/L)**	1509.1 ± 4868.9
**Albumin mean ± SD (g/L)**	38.4 ± 5.02
**Total Bilirubin (µmol/L)**	23.3 ± 30.46
**Aspartate aminotransferase (AST) (U/L)**	67.3 ± 60
**Alanine aminotransferase (ALT) (U/L)**	66.23 ± 63.51
**Hemoglobin (g/L)**	132.17 ± 22.23
**Leucocyte count (×10**^**9**^**/L)**	6± 1.5
**Platelet count (×10**^**9**^**/L)**	158.52 ± 81.2
Performance (ECOG* scale)	0:1 (12:5)
**Baseline Tumor Volume (ml) mean ± SD**	240 ± 210

### SIRT treatment

Liver vessels were mapped, and extrahepatic shunting was evaluated through angiography. A patient-specific treatment plan was developed based on voxel-wise dosimetry from ^99m^Tc-macroaggregated albumin (^99m^Tc-MAA) SPECT/CT images as a surrogate of ^90^Y microspheres. Lung shunt fraction (LSF) was calculated based on planar images. Dosimetry and treatment planning was conducted using Simpliciti90Y^TM^ treatment planning system (Mirada Medical Ltd, United Kingdom). The median of administered activity of ^99m^Tc-MAA was 156 MBq [range: 133–181]. After an interval of 32.8 ± 20.25 days, a median activity of 2.8 GBq [range: 1.24–6.3 GBq] of ^90^Y microspheres was administered to the same vessels through catheterization and the residual activity inside the vials was measured.

### Image analysis

Multi-phasic contrast-enhanced CT on the SOMATOM Definition Edge (Siemens Healthineers, Erlangen, Germany) or MR images performed on 3T Magnetom Skyra (Siemens Healthineers, Erlangen, Germany, ) were acquired almost a month before simulation (baseline). SPECT/CT images were acquired right after simulation or treatment on a dual-head Symbia-T series camera (Siemens Healthineers, Erlangen, Germany) by imaging ^99m^Tc photons with [128–150] keV energy window using a low-energy, high-resolution collimator, a 128 × 128 matrix, 64 views, over a 180-degree arc and 20–25 s per view. SPECT/CT data were reconstructed using 3D-ordered-subset expectation maximization (3D-OSEM) algorithm with 4 iterations and 8 subsets, while scatter correction was off, attenuation correction was on, and a gaussian postprocessing filter of 5 mm was applied. The images of ^90^Y-bremsstrahlung photons were acquired under the same scanner and parameters with the following differences: continuous energy window [105–195] keV using high energy collimator, and 64 views with 15–30 s per view.

### Dosimetry

The target volumes (tumors) were segmented on diagnostic images and the perfused lobe was delineated on the co-registered attenuation correction CT (ACCT) of SPECT/CT images by an experienced nuclear medicine specialist. Tumor segmentations were transferred on the SPECT/ CT images through registration of diagnostic images and co-registered CT of SPECT/CTs using elastix toolbox through a rigid registration followed by a deformable registration transform. The whole liver was segmented using a previously trained deep learning model with a dice factor of ~ 97% [38] on ACCT of SPECT/CT images, followed by visual check and modification, if necessary.

We calculated the physical as well as the biological effective dose (BED) maps using both ^99m^TC-MAA simulation and ^90^Y therapy for each patient as follows:

3D Voxel-wise physical dose maps were calculated according to the Local Deposition Method (LDM, denoted as physical or Dose in this study) by applying an in-house MATLAB code (MATLAB (2022b), Natick, Massachusetts: The MathWorks Inc) validated against replicated analysis with Simplicit90Y™ (Mirada Medical, United Kingdom).

The voxel-wise BED maps were calculated separately for tumor and for healthy structures (normal perfused liver (NPL) and whole normal liver (WNL)) by converting the physical absorbed dose of each voxel to BED using:

1 $$BED=D\left(1+\frac{D}{{~}^{\alpha }\!\left/ \!{~}_{\beta }\right.} . \frac{{T}_{Rep}}{{T}_{Rep}+{T}_{phys}}\right)$$

where D is the cumulative dose of ^90^Y radiation, T_Rep_ is the sublethal damage repair half-time and T_phys_ is the radionuclide decay half-life (64.2 h). ${~}^{\alpha }\!\left/ \!{~}_{\beta }\right.$ ratios were set to 10 for both normal and tumoral tissues, whereas T_Rep_ (h) was set to 1.5 and 2.5 for Tumoral and Normal structures, respectively. These values were derived from a radiobiological study on glass-microsphere SIRT by Chiesa et al. [39].

The bin size was set to 0.1 Gy to construct dose volume histograms (DVHs). We calculated the Mean absorbed dose, maximum, minimum, DVCs obtained from DVHs including D_50_, D_70_, D_95_, D_98_, V_20_, V_30_, V_50_, V_70_, V_90_, V_120_, V_205_, V_400_ (D_x_: minimum dose received by x% of the volume; V_x_: the percentage of the volume receiving at least x Gy) as recommended by a body of evidence[33, 40–42], for three defined volumes of interest including Tumoral liver (TL), NPL, WNL. The two laters are obtained from subtracting the TL from perfused liver and whole liver structures, respectively. Also, tumor to normal liver ratio (TNR) with respect to the mass of NPL (TNR_NPL_), and WNL (TNR_WNL_) was calculated for both simulation and therapy [40]. We also introduced a simplified homogeneity index (HI) borrowed from EBRT; defined as D_5_/D_95_ of tumor [43]. Other dosimetry related parameters such as ^90^Y-injected activity and lung shunt fraction and volume of each structure were included among the features. All dosimetry metrics were calculated once for physical dose maps and once for Biologically effective dose maps.

### Endpoints and strategy designing

The endpoints of this study were defined as the time from treatment procedure to death by any cause for OS, and the time from treatment procedure to local disease progression or development of metastasis for PFS.

The biomarkers and dosimetry parameters were considered as predictive features of the OS and PFS endpoints. We designed four distinct strategies for each of the two endpoints, resulting in a total of eight strategies. These strategies are denoted as “Clinical,” “Simulation”, “Therapy,” and “All Features”. The Clinical Strategy is comprised of a total of 16 clinical features. Simulation and therapy strategies include dosimetry features derived from physical and BED dose maps, calculated based on ^99m^Tc-MAA SPET/CT and ^90^Y SPECT/CT images, respectively. Additionally, both strategies include tumor volume, NPL, and WNL as shape features. All Features Strategy integrates all 305 features utilized across the Clinical, Simulation, and Therapy strategies. The complete name and description of the features are provided in Supplemental-Table 1.

### Univariate analysis

To assess the impact of each individual featured on OS and PFS outcomes, a univariate analysis was conducted using Cox proportional hazard models. The Cox models were fitted for each feature to calculate the hazard ratios (HR) and the results were subsequently evaluated using the Wald statistical test. The threshold for statistical significance was set as p-value < 0.05. Additionally, for each feature the concordance index (C-Index) along with corresponding standard errors were calculated. The Kaplan-Meier (KM) curves were constructed for each individual feature. Log-rank test (significance threshold: p-value < 0.05) was used to evaluate the statistical significancy of the observed differences in survival distribution. The median of each continuous feature was considered as the cut-off for group stratification. Noteworthy, the non-usual DVCs such as V_30_(%)-tumor or D_95_(Gy)-WNL were not used for univariate analysis.

### Feature selection and machine learning modeling

A cross-combination of various supervised ML algorithms and feature selection (FS) methods were analyzed. To identify the most relevant predictors and eliminate redundant features, we employed five different FS methods: univariate C-Index (UCI), Minimal Depth (MD), Mutual Information (MI), Variable hunting (VH), Variable hunting Variable Importance (VH. VIMP).

Using each FS method, No more than seven features were selected based on the recommendation of having a minimum ten observations per feature [44]. The redundant features were removed using Spearman’s correlation test. Pairs of features with a Spearman’s rank correlation coefficient exceeding 0.95 (rho > 0.95) were identified as redundant pairs, and subsequently one from each pair was removed.

The performance of five ML algorithms was evaluated: Cox Proportional Hazard regression (CoxPH), Generalized Linear Model Boosting (GLMB), Generalized Linear Model Network (GLMN), Random Survival Forest (RSF), and Survival Tree (ST). Further details about the FS and ML algorithms utilized in this study can be found in the Supplemental ML section as well as in [45, 46].

We adopted 3-fold cross-validation (CV), whereby there is a combination of inner and outer CV loops. In the inner CV, the dataset was divided into three folds and the model trained on different combinations of training and validation sets. The process was iterated and then the optimal hyperparameters were selected based on the calculated C-indices in the inner CV loop. The outer CV loop was used for estimating the model’s performance; the data were split into three-fold and for each fold, the model was trained on the training dataset using the most effective hyperparameters. This process was iterated for each fold in the outer CV loop, and the C-indices averaged to provide a more robust estimation of the model’s generalization performance. We used this approach to obtain a more reliable performance and to prevent overfitting the hyperparameters to a specific dataset. It should be mentioned that prior to FS and modeling, the features were normalized to their Z-Score based on the training dataset and the same transformation applied to the test dataset of the outer CV loop. Then, FS was performed on the training dataset of the outer CV loop, and the selected features were fed into each model.

**Fig. 1:**
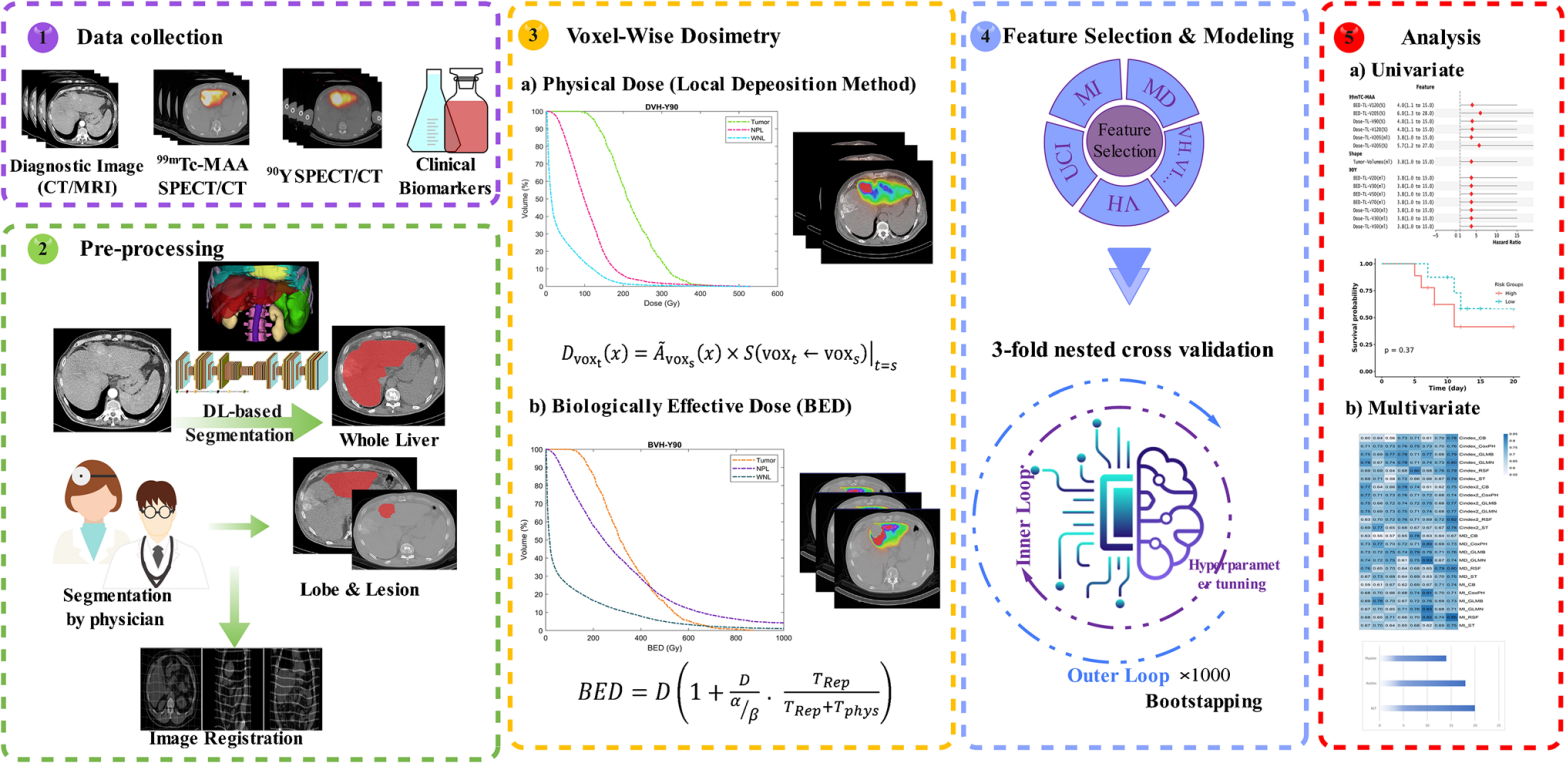
Flowchart of the methodology followed in this study protocol

In addition, bootstrap aggregation strategy (with 1000 bootstrap samples, with replacement) was implemented to compensate for small data size and prevent overfitting. The Wilcoxon signed-rank statistical test was employed to compare the predictive capabilities of each model. A p-value < 0.05 was considered significant. FS, modeling, and KM curves were calculated using R package (version 4.1). Figure 1 summarizes the adopted methods

## Results

### Study population characteristics

Among all 17 participants in this study, no severe adverse events were recorded. Predominantly, patients exhibited partial responses according to the modified Response Evaluation Criteria in Solid Tumors (mRECIST) criteria. Among the 17 patients included in this study, two patients underwent lobectomy by surgery after 7- and 8-months post-SIRT and were subsequently lost to more follow-up.

The median OS was eleven months with a 95% CI of [8.5, 13 months], ranging from 5 to 20 months. Median PFS was seven months with a 95% CI of [5.6, 10.9 months], ranging from 3 to 20 months.

All patients were followed-up on average 10.8 months (range 6–20 months) after treatment. Two patients failed to follow-up after 7–8 months as they underwent surgical lobectomy and fell out of the follow-up for this treatment. Over the course of 20-months follow up, seven patients passed away, leaving ten cases still alive. Additionally, ten cases developed metastasis or local progression, leaving seven cases free from disease progression. Among ten patients who showed progression after treatment wherein metastasis in the adrenal gland, lymph nodes, subcutaneous and liver metastasis and 6 cases of local progression were observed.

### Dosimetry results

The median ± SD of the physical and biologically effective dosimetry parameters for tumors and OARs (NPL and WNL) from both ^99m^Tc-MAA and ^90^Y are provided in Supplemental-Table 2. The median of LSF was 4.3% [range: 0.8–16.9%]. The median of tumoral mean absorbed doses ± SD were 345.6 ± 179.6, and 662.95 ± 521.65 Gy for ^99m^Tc-MAA physical and BED, respectively. For ^90^Y, the median of tumoral mean absorbed doses ± SD were 256.23 ± 143.66 and 413.78 ± 583.73 Gy for physical and BED, respectively.

**Fig. 2:**
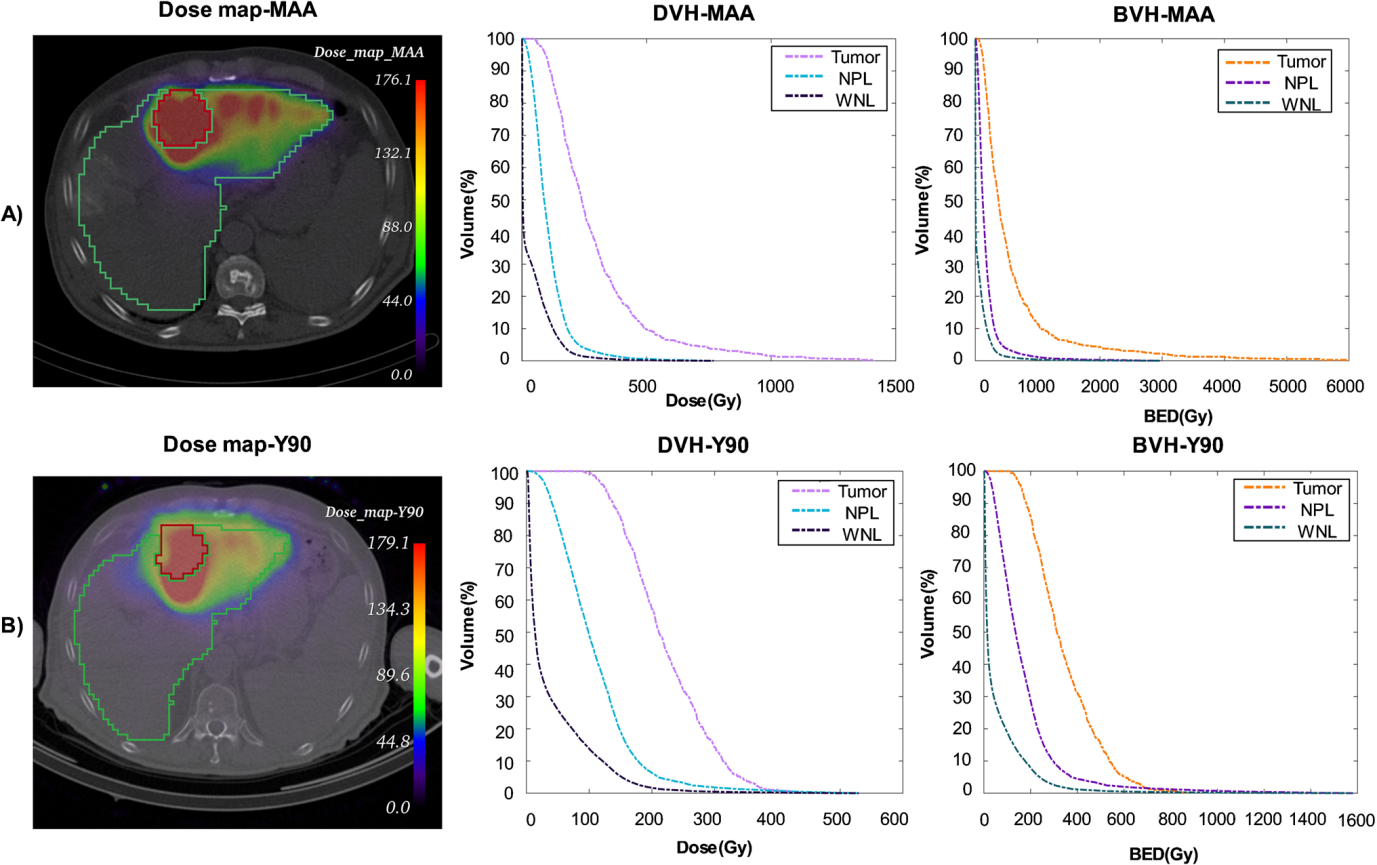
A 67-year-old male patient diagnosed with HCC who received 2.39 GBq of ^90^Y as palliative treatment. His OS and PFS were recorded as 6 months. Panel (**A**) depicts an axial slice of ^99m^Tc-MAA treatment planning dose map along with the corresponding extracted DVH and BVH (DVH form BED). The lesion and normal liver are delineated in red and green, respectively. Panel (**B**) shows the same information from ^90^Y treatment verification

A representative clinical example of physical dose maps and corresponding DVH and BVH (DVH from BED) for ^99m^Tc-MAA and ^90^Y is demonstrated in Fig. 2.

### Univariate analysis

**Fig. 3:**
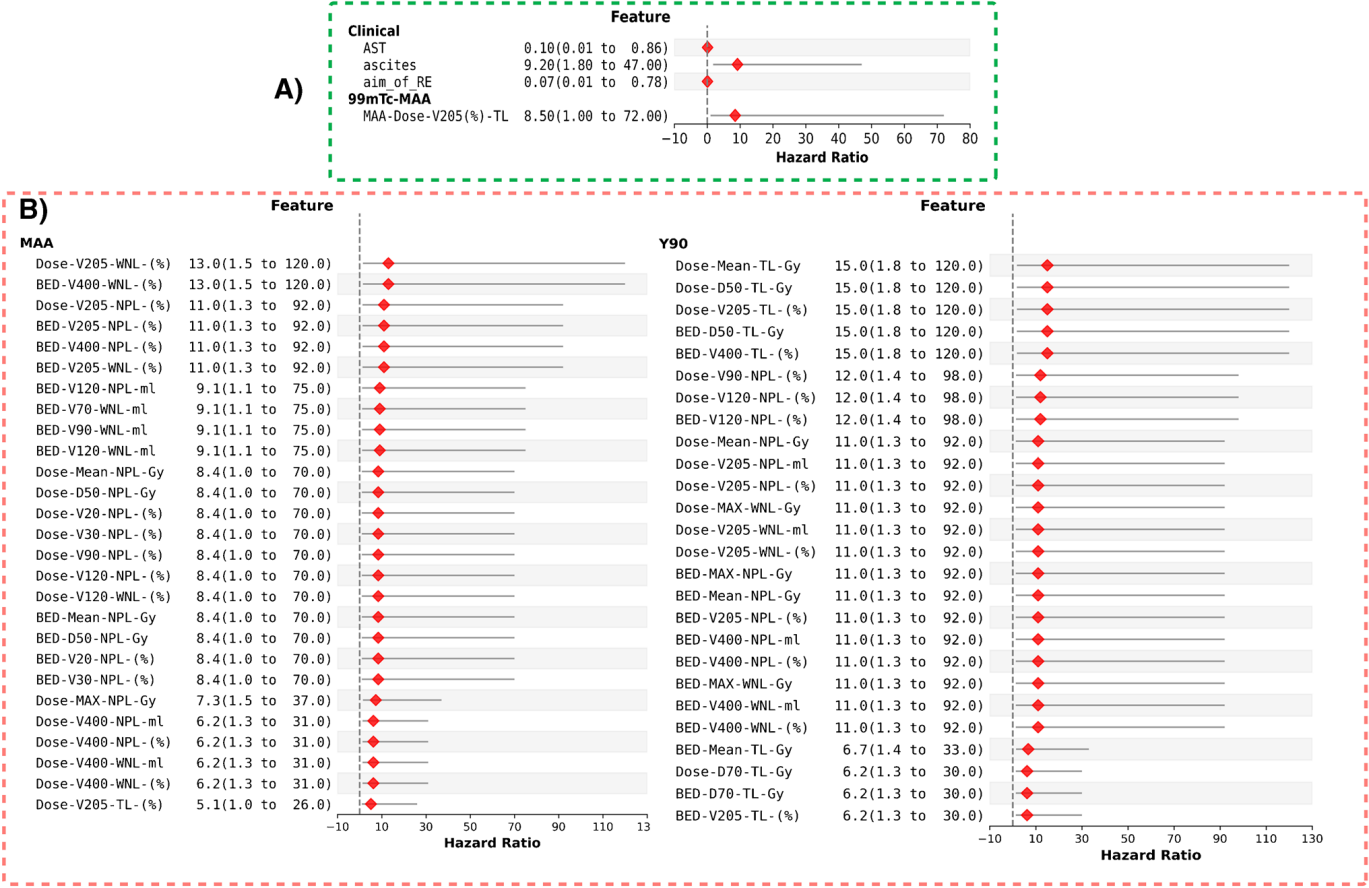
Cox proportional hazard ratios for each statistically significant feature predicting (**A**) OS and (**B**) PFS. HR > 1 is indicated in red, and the confidence interval is shown as a gray line

Figure 3 displays a forest map presenting hazard ratios for statistically significant features (p-value < 0.05) determined through univariate Cox proportional hazards analysis. The results for OS and PFS are reported in the following sections below.

### Univariate analysis-OS

Notably, among the clinical features, the presence of “Ascites” revealed a substantial association with OS (HR = 9.2, 95% CI: [1.8, 47]), indicating a significantly higher risk in the Ascites group compared to those without Ascites.

Within the ^99m^Tc-MAA dosimetry features, “MAA-Dose-V_205_(%)-TL” demonstrated a hazard ratio of 8.5, 95%CI: [1,72]. The comparison groups were stratified based on the median values of the features. Seemingly, patients whose 78% of their tumor was covered by at least 205 Gy demonstrated prolonged OS. Additional details on the univariate Cox proportional hazard models predicting OS can be found in Supplemental-Table 3.

### Univariate analysis-PFS

Based on this dataset, no clinical feature was recognized for predicting PFS through univariate analysis. Among ^99m^Tc-MAA dosimetry features, “MAA-Dose-V_205_(%)-WNL” and “MAA-BED-V_400_(%)-WNL” were strongly associated with PFS, showing a hazard ratio of 13 (95% CI: [1.5, 120]). Among ^90^Y-dosimetry features, “^90^Y-Dose-mean-TL(Gy)”, “^90^Y-Dose-D_50_-TL(Gy)”, “^90^Y-Dose-V_205_(%)-TL”, “^90^Y-BED-D_50_-TL(Gy)”, “^90^Y-BED-V_400_(%)-TL” emerged as statistically significant predictors for PFS, with the maximum HR of 15 (95% CI: [1.8, 120]). Patients whose tumors received a mean dose of 260 Gy or higher exhibited an extended PFS compared to those with a tumor dose (TD) less than 260 Gy. Details of the univariate Cox proportional hazard models predicting PFS are summarized in Supplemental-Table 4.

**Fig. 4.**
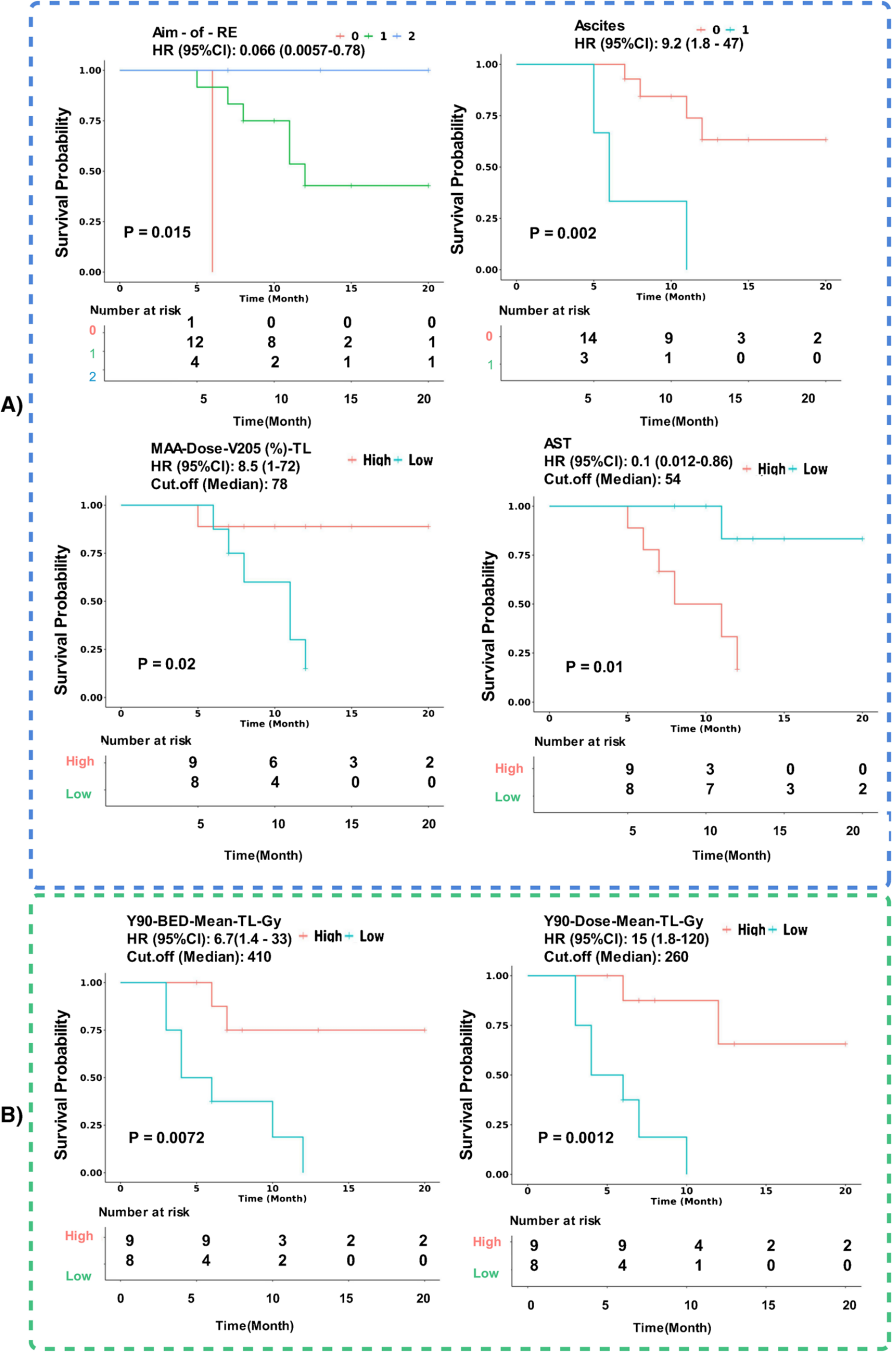
**Panel A)** The features associated with OS within the univariate analysis. The aim-of-RT means whether patient was treated as 0. Palliative, 1. Lobectomy and 2. segmentectomy. The presence of Ascites, with 0 and 1 legends indicating the non-presenting-ascites and ascites groups, respectively. The non-presenting-ascites group survived longer than ascites group. MAA-Dose-V_205_-TL (%): The group whose more than 78% of their tumors received at least 205 Gy survived more. Also, the group with Aspartate aminotransferase (AST) < 54 experienced more OS. **Panel B**) ^90^Y-tumoral mean dose values from physical and BED calculations associated with PFS; the individuals received mean dose ≥ 260 and mean BED≥ 410 Gy had a longer PFS

KM curves for all denoted features in Fig. 3 are provided in Supplemental-Figures 1 and 2 with stratification based on the median values of the continuous features. Figure 4 illustrates the KM curves for clinical and mean absorbed dose features predicting OS, as well as the tumoral mean doses that found statistically meaningful for predicting PFS. To optimize the stratification of these features, we also computed the optimal cut points that yielded the most distinct (stratified) KM curves. The corresponding KM curves based on these optimal cut points can be found in Supplemental-Figure 3. The optimal cut points for Aspartate aminotransferase (AST), MAA-Dose-V_205_-TL (%), ^90^Y-Dose-mean-TL(Gy) and ^90^Y-BED-mean-TL(Gy) were 55 (U/L), 75.2%, 244.7 Gy and 413.78 Gy, respectively.

### Machine learning

**Fig. 5:**
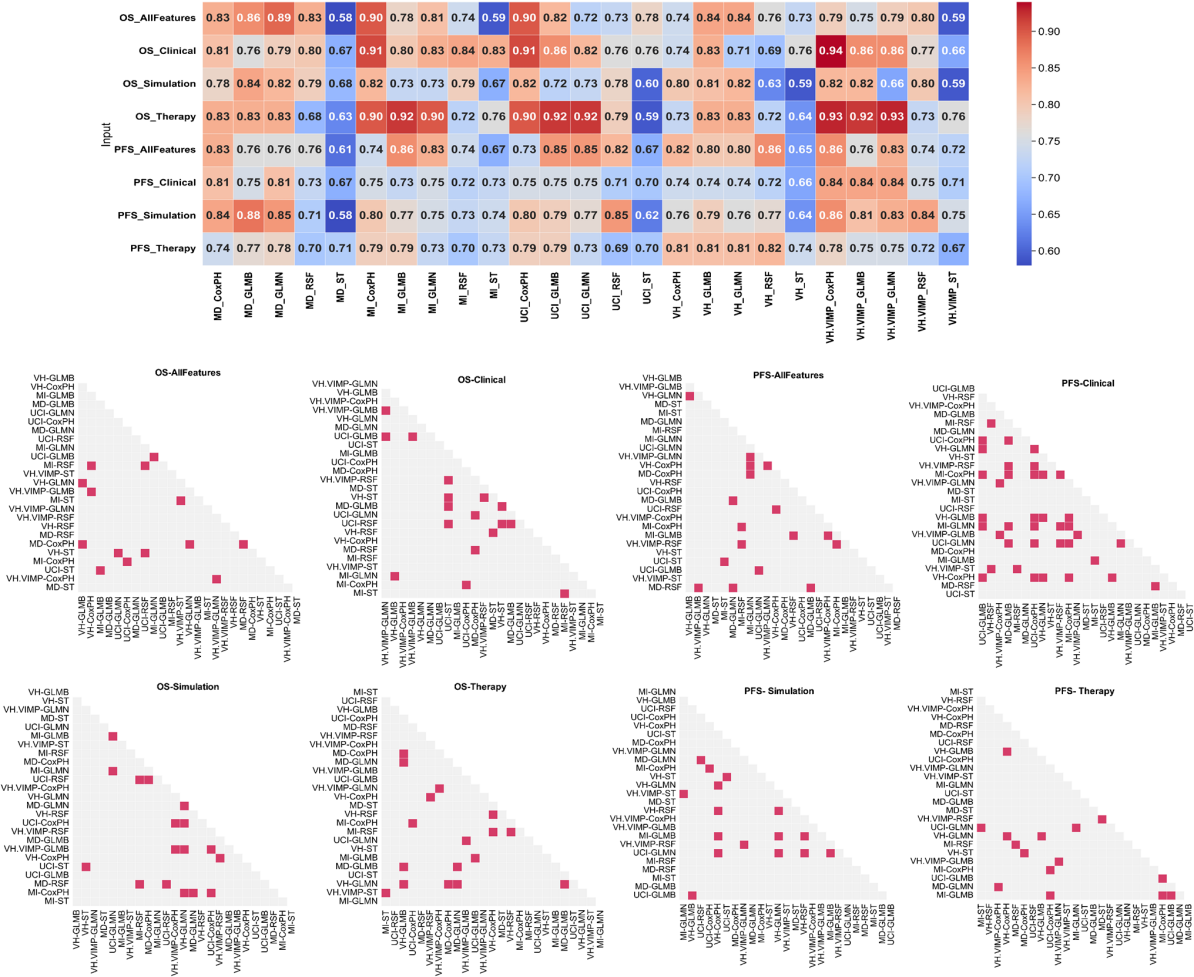
Heat map of mean C-indices obtained from all cross combinations of feature selection and machine learning models. On the right side, the plots of p-values calculated from Wilcoxon statistical test for each strategy. The red cells show non-significancy (*p*-value ≥ 0.05)

The heat map in Fig. 5 displays the mean C-indices resulting from an extensive analysis involving 25 cross-combinations of feature selection and ML models. Accompanying this visualization are the plots showing the results of Wilcoxon statistical tests (p-value) for each strategy. Optimal prognostic models were identified based on both high performance (highest C-indices) and statistical significance. Table 2 summarizes the C-indices, SD, and confidence intervals (CIs) for these selected models.

**Table 2:** Optimal prognostic models for predicting OS and PFS within each strategy presenting statistically significant differences than the others. The table includes the mean Concordance Index (C-index) accompanied by the standard deviation (SD) and confidence interval (CI) for comprehensive assessme﻿nt

	OS				PFS			
	Model	C-Index	SD	CI	Model	C-Index	SD	CI
All Features	MI-CoxPH UCI-CoxPH	0.9 0.9	0.16 0.16	0.89–0.91 0.89–0.9	MI-GLMB VH-RSF VH. VIMP-CoxPH	0.86 0.86 0.86	0.14 0.14 0.17	0.85–0.86 0.85–0.86 0.85–0.87
Clinical	VH. VIMP-CoxPH	0.94	0.13	0.93–0.94	VH. VIMP-CoxPH VH. VIMP-GLMB VH. VIMP-GLMN	0.84 0.84 0.84	0.16 0.16 0.16	0.84–0.85 0.84–0.85 0.84–0.85
Simulation	MD-GLMB	0.84	0.18	0.83–0.84	MD-GLMB	0.88	0.16	0.88–0.89
Therapy	VH. VIMP-CoxPH VH. VIMP-GLMN	0.93 0.93	0.14 0.14	0.93–0.94 0.93–0.94	VH-RSF	0.82	0.17	0.82–0.83

The KM curves were constructed to visualize the performance of the selected models. The median of the models’ risk scores served as the cut-off for stratification, and the Log-rank test was applied to assess the statistical significance.

**Fig. 6:**
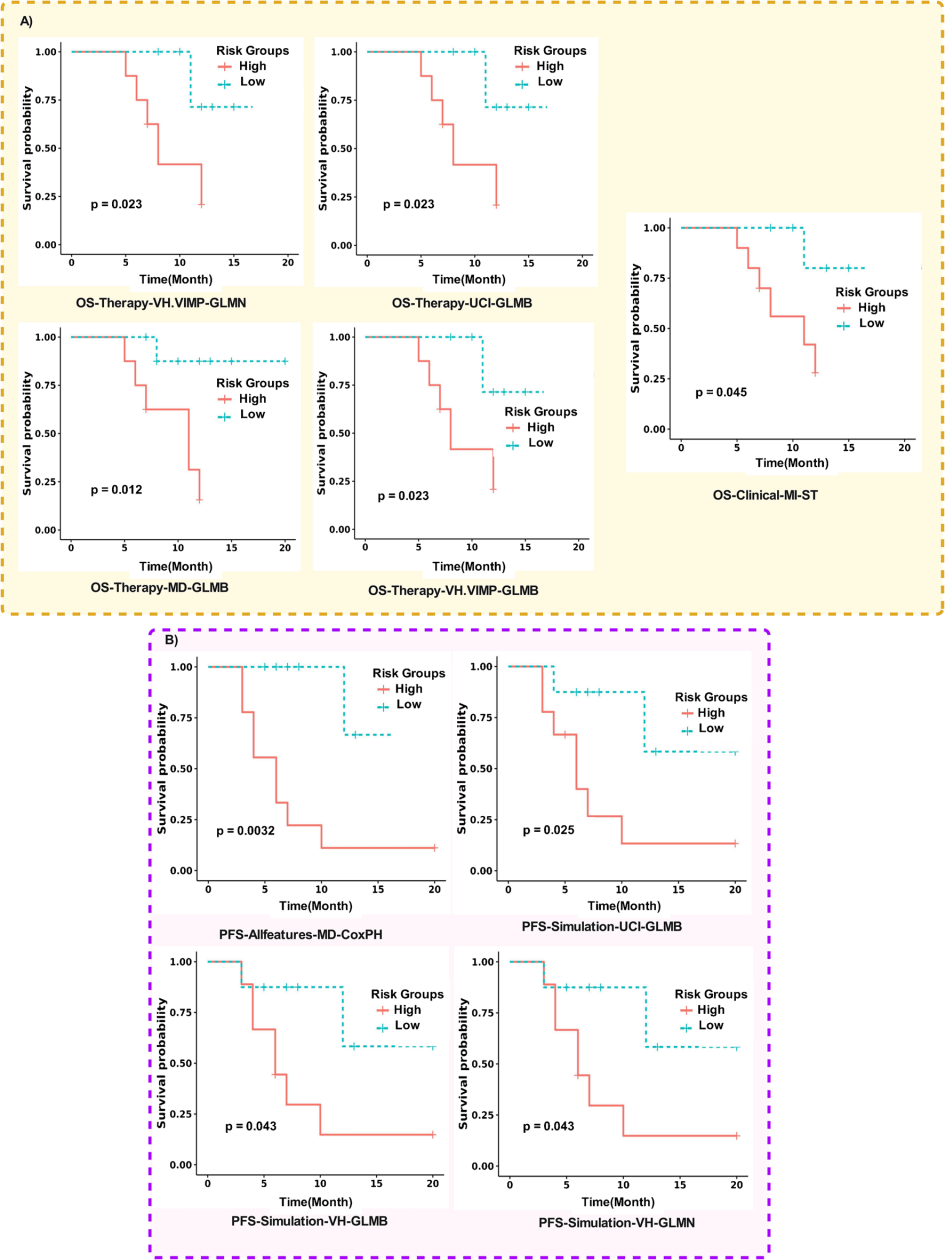
The KM curve for models predicting **A** “OS” and **B** “PFS” with statistically significant stratification between high and low-risk groups. The calculated median of the risk scores by the models was used as cut-off criteria for stratification. The high-risk individuals had a risk score above or equal to median while the low-risk groups had a risk-score below the median. The p-values were calculated by log-rank statistical test

The “OS-Therapy-VH.VIMP-GLMN” model exhibited one of the highest C-index values and demonstrated a statistically significant KM curve. Figure 6 shows the KM curves for models that efficiently stratified patients into two groups, regardless of their performance in terms of C-index. Additionally, we provided the KM curves for the selected high C-index models in Supplemental Fig. 4.

**Fig. 7:**
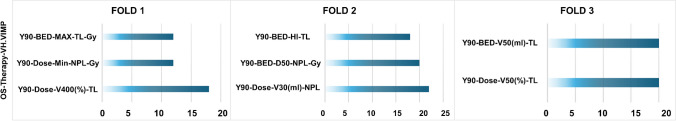
Illustration of selected features within each fold, and their importance calculated for the VH. VIMP-GLMN model predicting the OS in Therapy strategy

In Fig. 7, the selected features within the three folds contributing to the predictive power of the OS-Therapy-VH.VIMP-GLMN model are presented. The selected features through each FS method in each strategy are outlined in Supplemental-Tables 6–9 for OS and Supplemental-Tables 10–13 for PFS. Supplemental Figures 5 and 6 depict the feature importance of the selected features, contributing to constructing the efficient (selected) models. 

## Discussion

Early and accurate prediction of OS and PFS in patients undergoing SIRT procedure is important and can serve as a crucial guide for optimal patient selection, aiding in considering alternative treatments when necessary. The utilization of dosimetry parameters, specifically derived from voxel-level dose maps, contributes to the assessment of treatment planning and verification. These parameters offer insights into the status of tumors and OARs. While the impact of clinical biomarkers and mean absorbed dose in tumor response and OS has been explored through statistical models, there remains a notable gap in understanding the role of other potentially valuable dosimetry parameters in predicting outcomes. Further investigation is imperative to uncover the full spectrum of benefits these features may offer in enhancing predictive models for patient prognosis. Notably, as artificial intelligence plays a pivotal role in medical decision-making, the development of effective predictive models becomes more essential. Establishing robust models for outcome prediction not only facilitates decision-making processes but also enhances accuracy in prognosis. Hence, it’s important to thoroughly explore different dosimetry parameters and include them in detailed predictive models to predict patient outcomes.

We explored the impact of each baseline clinical biomarker, alongside the dosimetry parameters, derived from both physical and biological effective dose maps in treatment planning and verification of SIRT using statistical models. In parallel, 200 models were trained, incorporating various feature selection methods and ML algorithms. These models were designed to predict OS and PFS in four distinct strategies.

This study strategically utilized well-established features extracted from DVH and BVH of the ^99m^Tc-MAA simulation and ^90^Y treatment verification, instead of additional radiomic or dosiomic features requiring extra time and effort, given the challenges associated with the interpretability of many such features as well as reproducibility over different calculation parameters. Given the lack of spatial information in DVHs, this study does not include the spatial information and the locations where inhomogeneities occur, the information that radiomic and dosiomic features may provide. For instance, we used a homogeneity index defined in EBRT, whose appropriateness for SIRT should be assessed. However, there are some radiomic features that may be more beneficial in this context.

Different scenarios were considered, including the use of only simulation or therapy dosimetry features, only clinical biomarkers, or a comprehensive set of all features combined. This study design allows us to explore and evaluate distinct sets of features tailored to each strategy, leading to understanding of their respective impacts on the study’s endpoints.

To the best of our knowledge, this study is the first study using ML in conjunction with various dosimetry parameters derived from both simulation and therapy sessions. This approach aimed to predict the OS and PFS outcomes in patients undergoing ^90^Y-SIRT. We extended our analysis to include dosimetry features extracted not only from tumors but also from healthy organs (NPL and WNL). This approach was inspired by a recent study that highlighted the importance of radiomics features from both tumors and organs at risk for predicting OS [46]. To date, a study on SARAH trial dataset indicated that the tumor mean-absorbed-dose (with cutoff of 100 Gy using resin microspheres) from MAA is an independent predictor of prolonged survival [36]. Using glass microspheres, our univariate analysis has unveiled the potential of several features capable of independently predicting OS and PFS (see Fig. 3 and Supplemental Figures 1–2). Our research indicated a mean absorbed dose of ≥ 260 Gy to the tumor, calculated based on ^90^Y, as a threshold of predicting PFS while in the literature almost the same value was reported for predicting OS [31, 47, 48]. No corresponding value was found as a threshold based on MAA- dosimetry. Nevertheless, it’s crucial to note that the threshold identified in this study should not be regarded as threshold for an optimal clinical outcome.

DVHs are widely used to summarize and quantify dose distribution information, by visually depicting the distribution. However, the granularity of a DVHs depends on the selected dose-volume bin size, and slight changes in bin size can change the shape of the DVH curve. This matter could potentially influence the extracted DVC parameters and the interpretations [49, 50], which should be considered. We used an empirical bin size of 0.1 Gy to consider the minimum detectable changes within a dose distribution. It should be emphasized that we conducted volume-constraint calculations based on both percent and milliliter (ml). The percentage-based approach provides a sense of normalized volume to the total volume. Additionally, we adopted the median of these features as the threshold for stratifying KM curves. For certain features, such as mean absorbed dose values, we adopted the optimal thresholds instead of median. The first (median cutoffs) approach yielded a more balanced distribution of the population between the two groups. Whereas, identifying optimal cutoffs for certain features led to generating an unbalanced distribution.

We evaluated the role of baseline laboratory values, such as albumin, bilirubin, Alpha fetoprotein (AFP),  Portal vein thrombosis (PVT), etc. as significant factors for predicting OS [22, 24]. There was no statistically meaningful correlation between AFP or PVT and the endpoints based on our univariate results. However, these features were selected multiple times through feature selection methods demonstrating their predictive importance (Supplemental-Tables 6–13).

Baseline clinical features, including Ascites (log-rank p-value of 0.002) and the aim of Radioembolization, and AST levels were found to be statistically meaningful prognosticators for OS. The aim of Radioembolization including segmentectomy, lobectomy or palliative groups showed a longer OS for segmentectomy group (log-rank p-value = 0.015). An AST level of 54 U/L stratified the KM curves (log-rank p-value = 0.01). The other important baseline clinical prognosticators could be e.g., Barcelona Clinic for Liver Cancer (BCLC), Child Pugh, and performance status (e.g. ECOG). Information regarding BCLC and Child Pugh was missing due to the retrospective nature of the study, and no correlation was observed between ECOG and both endpoints. Additionally, tumor response could serve as an important predictor of survival. However, it was not included as a feature both in univariate and into the models, given the lack of standardized approach for response assessment. Moreover, the primary objective of the study was early prediction of the endpoints using baseline clinical or dosimeric parameters, preferably prior to treatment or based on treatment verification images, excluding follow-up images. Moreover, the impact of tumor response in predicting such outcomes could be found elsewhere [25].

The α/β ratios, which are essential radiosensitivity parameters used for BED calculations in this study, have been sourced from the study by Chiesa et al. [39] where a tumor control probability (TCP) model was developed to extract α values. However, it’s important to note that studies focusing on TCP modeling and biologically effective dosimetry for SIRT are scarce. Many of the studies have utilized values derived from EBRT, which is a fundamentally distinct treatment from SIRT [42]. The EBRT-adopted α and β values are derived from empirical analyses of linear quadratic models, incorporating uncertainties [51]. Therefore, given the inherent differences between EBRT and SIRT, thorough studies on TCP modeling, BED calculations and radiosensitivity parameters are still required.

Our multivariate analysis by ML demonstrated the feasibility of predicting the endpoints using the introduced features. Cross-combination of 25 models and FS methods for 8 strategies resulted in a total of 200 models. The performance of these models was systematically evaluated through the average C-index on a 3-fold CV. Publicly available ML algorithms were selected. They proved to be capable of handling the continuous time-to-event survival data. The feature selection methods were applied to select the most relevant features and to improve the performance of the models. It should be noted that, in the multivariate analysis, we used all the extracted features prior to feature selection, while some of the features might not be evaluated in current (clinical and research-based) dosimetry calculations (e.g. V_30_ of tumor or D_95_ of WNL). Nonetheless, we used them all and let the FS algorithms decide on keeping the feature or identifying it as irrelevant or redundant. Noteworthy, for univariate analysis, we did not utilize such features.

While, for each strategy, at least one model outperformed the others in terms of the C-Index (Table 2), we constructed KM curves to validate the overall model performance. Notably, among the KM curves, only one model (OS-Therapy-VH.VIMP-GLMN, C-index = 0.93) that predicted OS using Therapy strategy features effectively stratified the high and low-risk groups (log-rank p-value = 0.023). Although the OS-Clinical-VH.VIMP-GLMN model’s C-index was 0.94, i.e. higher than VH.VIMP-GLMN, this model failed to stratify the groups within KM curves. To explain this phenomenon, C-index assesses how well patients are ranked based on their risk of experiencing an event, whereas the KM curve shows actual survival probabilities over time. While models with high C-index values may effectively rank patients, they may not accurately estimate survival probabilities at specific time points. Therefore, we evaluate models using multiple metrics to ensure performance in different aspects. Limited data may contribute to this discrepancy, as models may excel at calculating the C-index but struggle to predict survival dynamics accurately.

To ensure that ML models’ decision-making procedures are explainable, it’s crucial to clarify the contribution of each individual input feature [52]. Therefore, we provided the importance of the features selected and used by the efficient models (Fig. 7 and Supplemental-Figures 5 and 6).

While our study provided valuable insights, caution should be taken in interpreting the results due to the inherent limitations. The retrospective nature of the study limited our access to only a subset of clinical data, excluding variables, such as Child Pugh and tumor staging, etc. The limited sample size collected from a single center further restricted us in compromising the robustness of the ML models. Another limitation is the lack of external validation. Unfortunately, we did not have access to any clinical database that can serve as external validation dataset to evaluate the robustness of our models on data from other centers with different dose calculation schemes, scanning protocols and population demographics. Moreover, the presence of right censoring in the dataset implies that not all patients had sufficient follow-up time to experience the events of interest. Additionally, the relatively short duration of the follow-up period represents another limitation. Therefore, more investigation on large-scale dataset collected from multiple centers with a long-enough follow-up period is still required.

Although we utilized SPECT images corrected for physical degrading factors, it should be emphasized that the drawbacks of SPECT imaging, such as limited spatial resolution and high noise, effects of physical degrading factors such as attenuation, scatter, and collimator septal penetration etc. may affect the dosimetry results. The images should be corrected for the degrading factors prior to dosimetry calculations. Nevertheless, PET/CT images are more suitable for dosimetry and further studies.

All in all, developing models with large-scale and multiscale dataset, with reproducible results, enables clinicians to tailor individualized treatment strategies and optimize patient care. Identifying patients with good/poor prognosis may affect clinician’s decisions in treatment and follow-up regimen. Patients presenting with good prognosis may be considered for less aggressive treatments or less frequent monitoring, while patients presenting with poor prognosis may benefit from more intensive therapeutic interventions or more frequent follow-ups.

## Conclusion

The feasibility of developing ML models to predict survival outcomes using dosimetry parameters obtained from both ^99m^Tc-MAA and ^90^Y SPECT images, was evaluated. The findings are based on a personalized dosimetry-based dataset that can contribute not only to the refinement of predicting outcomes for SIRT but also has the potential to pave the way for the establishment of dose-effect relationship in SIRT treatment.

## Supplementary Information

Below is the link to the electronic supplementary material.

Supplementary file1 (PDF 1.83 MB)

## Data Availability

The data used in this work is not available.

## Abbreviations

TL: Tumoral liver
NPL: Normal perfused liver
WNL: Whole normal liver
OS: Overall survival
PFS: Progression Free survival
DVH: Dose volume histogram
DVC: Dose volume constraints
ML: Machine learning
FS: Feature selection
CV: Cross validation
SIRT: Selective internal radiation therapy
SPECT/CT: Single photon emission/ computed tomography
PET/CT: Positron emission tomography/computed tomography
MAA: Macro aggregated albumin
OAR: Organ at risk
EBRT: External beam radiation therapy
BED: Biologically effective dose
HCC: Hepatocellular carcinoma
NET: Neuro endocrine Tumor
RE: Radioembolization
AST: Aspartate aminotransferasie
ALT: Alanine aminotransferase
AFP: Alphafeto protein
LSF: Lung shunt fraction
3D-OSEM: 3D-ordered-subset expectation maximization
ECOG: Eastern cooperative oncology group
ACCT: Attenuation corrected computed tomography
MAD: Mean absorbed dose
TNR: Tumor to normal ratio
UCI: Univariate C-Index
MD: Minimal Depth
MI: Mutual Information
VH: Variable hunting
VH. VIMP: Variable hunting Variable Importance
CoxPH: Cox Proportional Hazard regression
GLMB: Generalized Linear Model Boosting
GLMN: Generalized Linear Model Network
RSF: Random Survival Forest
ST: Survival Tree
CI: Confidence interval
C-index: Concordance Index
SD: Standard deviation
SARAH: Sorafenib versus Radioembolization in Advanced Hepatocellular Carcinoma
