# Supplementary material for: The role of biomarkers and dosimetry parameters in overall and progression free survival prediction for patients treated with personalized 90Y glass microspheres SIRT: a preliminary machine learning study
Source: Eur J Nucl Med Mol Imaging. 2024 Jul 9;51(13):4111–26. doi: 10.1007/s00259-024-06805-8 (PMC11639191; doi:10.1007/s00259-024-06805-8)
Supplement: Supplementary file 1 — Supplementary file1 (PDF 1.83 MB) [file 259_2024_6805_MOESM1_ESM.pdf]

Supplemental file

The role of biomarkers and dosimetry parameters in overall and progression free survival prediction for patients treated with personalized ^{90}Y glass microspheres SIRT: A preliminary machine learning Study

Zahra Mansouri[1], Yazdan Salimi[1], Ghasem Hajianfar[1], Nicola Bianchetto Wolf[1], Luisa Knappe[1], Genti Xhepa[2], Adrien Gleyzolle[2], Alexis Ricoeur[2], Valentina Garibotto[1,3], Ismini Mainta[1] and Habib Zaidi[1,4,5,6†]

[1]Division of Nuclear Medicine and Molecular Imaging, Diagnostic Department, Geneva University Hospital, Geneva, Switzerland

[2]Service of Radiology, Geneva University Hospital, CH-1211, Geneva, Switzerland

[3]Centre for Biomedical Imaging (CIBM), Geneva, Switzerland

[4]Department of Nuclear Medicine and Molecular Imaging, University of Groningen, University Medical Center Groningen, Groningen, Netherlands

[5]Department of Nuclear Medicine, University of Southern Denmark, Odense, Denmark

[6]University Research and Innovation Center, Óbuda University, Budapest, Hungary

Corresponding author
Habib Zaidi, Ph.D†
Geneva University Hospital, Division of Nuclear Medicine and Molecular Imaging, CH-1211 Geneva, Switzerland
Tel: +41 22 372 7258
Fax: +41 22 372 7169
Email: habib.zaidi@hcuge.ch

Table 1. Features adopted in this study along with their definitions.

NUMBER	FEATURE	DESCRIPTION
**1**	Volumes (ml)-TL	**Volume of the Tumor (ml)**
**2**	Volumes (ml)-NPL	**Volume of the normal perfused liver (ml)**
**3**	Volumes (ml)-WNL	**Volume of the Whole normal liver (ml)**
**4**	MAA-BED-Max (Gy)-TL	**Maximum dose of the tumor from BED of MAA**
**5**	MAA-BED-Max (Gy)-NPL	**Maximum dose of the normal perfused liver from BED of MAA**
**6**	MAA-BED-Max (Gy)-WNL	**Maximum dose of the whole normal liver from BED of MAA**
**7**	MAA-BED-Mean Dose (Gy)-TL	**Mean dose of the tumor from BED of MAA**
**8**	MAA-BED-Mean Dose (Gy)-NPL	**Mean dose of the normal perfused liver from BED of MAA**
**9**	MAA-BED-Mean Dose (Gy)-WNL	**Mean dose of the whole normal liver from BED of MAA**
**10**	MAA-BED-Min (Gy)-TL	**Minimum dose of the Tumor from BED of MAA**
**11**	MAA-BED-Min (Gy)-NPL	**Minimum dose of the normal perfused liver from BED of MAA**
**12**	MAA-BED-Min (Gy)-WNL	**Minimum dose of the whole normal liver from BED of MAA**
**13**	MAA-BED-D50(Gy)-TL	**The biologically effective dose from MAA (Gy) received by the 50% of the tumor volume**
**14**	MAA-BED-D50(Gy)-NPL	**The biologically effective dose from MAA (Gy) received by the 50% of the NPL volume**
**15**	MAA-BED-D50(Gy)-WNL	**The biologically effective dose from MAA (Gy) received by the 50% of the WNL volume**
**16**	MAA-BED-D70(Gy)-TL	**The biologically effective dose from MAA (Gy) received by the 70% of the tumor volume**
**17**	MAA-BED-D70(Gy)-NPL	**The biologically effective dose from MAA (Gy) received by the 70% of the NPL volume**
**18**	MAA-BED-D70(Gy)-WNL	**The biologically effective dose from MAA (Gy) received by the 70% of the WNL volume**
**19**	MAA-BED-D95(Gy)-TL	**The biologically effective dose from MAA (Gy) received by the 95% of the tumor volume**
**20**	MAA-BED-D95(Gy)-NPL	**The biologically effective dose from MAA (Gy) received by the 95% of the NPL volume**
**21**	MAA-BED-D95(Gy)-WNL	**The biologically effective dose from MAA (Gy) received by the 95% of the WNL volume**
**22**	MAA-BED-D98(Gy)-TL	**The biologically effective dose from MAA (Gy) received by the 98% of the tumor volume**
**23**	MAA-BED-D98(Gy)-NPL	**The biologically effective dose from MAA (Gy) received by the 98% of the NPL volume**
**24**	MAA-BED-D98(Gy)-WNL	**The biologically effective dose from MAA (Gy) received by the 98% of the WNL volume**
**25**	MAA-BED-Homogeneity-index-TL	**The ratio of D5/D95 in Tumor from biologically effective dose calculated from MAA**
**26**	MAA-BED-V20(ml)-TL	**The volume of tumor (ml) that receives ≥ 20 Gy of BED calculated from MAA**
**27**	MAA-BED-V20(ml)-NPL	**The volume of NPL (ml) that receives ≥ 20 Gy of BED calculated from MAA**
**28**	MAA-BED-V20(ml)-WNL	**The volume of WNL (ml) that receives ≥ 20 Gy of BED calculated from MAA**
**29**	MAA-BED-V20(%)-TL	**The volume of tumor (m%) that receives ≥ 20 Gy of BED calculated from MAA**
**30**	MAA-BED-V20(%)-NPL	**The volume of NPL (%) that receives ≥ 20 Gy of BED calculated from MAA**
**31**	MAA-BED-V20(%)-WNL	**The volume of WNL (%) that receives ≥ 20 Gy of BED calculated from MAA**
**32**	MAA-BED-V30(ml)-TL	**The volume of tumor (ml) that receives ≥ 30 Gy of BED calculated from MAA**
**33**	MAA-BED-V30(ml)-NPL	**The volume of NPL (ml) that receives ≥ 30 Gy of BED calculated from MAA**
**34**	MAA-BED-V30(ml)-WNL	**The volume of WNL (ml) that receives ≥ 30 Gy of BED calculated from MAA**
**35**	MAA-BED-V30(%)-TL	**The volume of tumor (%) that receives ≥ 30 Gy of BED calculated from MAA**
**36**	MAA-BED-V30(%)-NPL	**The volume of NPL (%) that receives ≥ 30 Gy of BED calculated from MAA**
**37**	MAA-BED-V30(%)-WNL	**The volume of WNL (%) that receives ≥ 30 Gy of BED calculated from MAA**
**38**	MAA-BED-V50(ml)-TL	**The volume of tumor (ml) that receives ≥ 50 Gy of BED calculated from MAA**
**39**	MAA-BED-V50(ml)-NPL	**The volume of NPL (ml) that receives ≥ 50 Gy of BED calculated from MAA**
**40**	MAA-BED-V50(ml)-WNL	**The volume of WNL (ml) that receives ≥ 50 Gy of BED calculated from MAA**
**41**	MAA-BED-V50(%)-TL	**The volume of tumor (%) that receives ≥ 50 Gy of BED calculated from MAA**
**42**	MAA-BED-V50(%)-NPL	**The volume of NPL (%) that receives ≥ 50 Gy of BED calculated from MAA**
**43**	MAA-BED-V50(%)-WNL	**The volume of WNL (%) that receives ≥ 50 Gy of BED calculated from MAA**
**44**	MAA-BED-V70(ml)-TL	**The volume of tumor (ml) that receives ≥ 70 Gy of BED calculated from MAA**

**45**	MAA-BED-V70(ml)-NPL	**The volume of NPL (ml) that receives ≥ 70 Gy of BED calculated from MAA**
**46**	MAA-BED-V70(ml)-WNL	**The volume of WNL (ml) that receives ≥ 70 Gy of BED calculated from MAA**
**47**	MAA-BED-V70(%)-TL	**The volume of tumor (%) that receives ≥ 70 Gy of BED calculated from MAA**
**48**	MAA-BED-V70(%)-NPL	**The volume of NPL (%) that receives ≥ 70 Gy of BED calculated from MAA**
**49**	MAA-BED-V70(%)-WNL	**The volume of WNL (%) that receives ≥ 70 Gy of BED calculated from MAA**
**50**	MAA-BED-V90(ml)-TL	**The volume of tumor (ml) that receives ≥ 90 Gy of BED calculated from MAA**
**51**	MAA-BED-V90(ml)-NPL	**The volume of NPL (ml) that receives ≥ 90 Gy of BED calculated from MAA**
**52**	MAA-BED-V90(ml)-WNL	**The volume of WNL (ml) that receives ≥ 90 Gy of BED calculated from MAA**
**53**	MAA-BED-V90(%)-TL	**The volume of tumor (%) that receives ≥ 90 Gy of BED calculated from MAA**
**54**	MAA-BED-V90(%)-NPL	**The volume of NPL (%) that receives ≥ 90 Gy of BED calculated from MAA**
**55**	MAA-BED-V90(%)-WNL	**The volume of WNL (%) that receives ≥ 90 Gy of BED calculated from MAA**
**56**	MAA-BED-V120(ml)-TL	**The volume of tumor (ml) that receives ≥ 120 Gy of BED calculated from MAA**
**57**	MAA-BED-V120(ml)-NPL	**The volume of NPL (ml) that receives ≥ 120 Gy of BED calculated from MAA**
**58**	MAA-BED-V120(ml)-WNL	**The volume of WNL (ml) that receives ≥ 120 Gy of BED calculated from MAA**
**59**	MAA-BED-V120(%)-TL	**The volume of tumor (%) that receives ≥ 120 Gy of BED calculated from MAA**
**60**	MAA-BED-V120(%)-NPL	**The volume of NPL (%) that receives ≥ 120 Gy of BED calculated from MAA**
**61**	MAA-BED-V120(%)-WNL	**The volume of WNL (%) that receives ≥ 120 Gy of BED calculated from MAA**
**62**	MAA-BED-V205(ml)-TL	**The volume of tumor (ml) that receives ≥ 205 Gy of BED calculated from MAA**
**63**	MAA-BED-V205(ml)-NPL	**The volume of NPL (ml) that receives ≥ 205 Gy of BED calculated from MAA**
**64**	MAA-BED-V205(ml)-WNL	**The volume of WNL (ml) that receives ≥ 205 Gy of BED calculated from MAA**
**65**	MAA-BED-V205(%)-TL	**The volume of tumor (%) that receives ≥ 205 Gy of BED calculated from MAA**
**66**	MAA-BED-V205(%)-NPL	**The volume of NPL (%) that receives ≥ 205 Gy of BED calculated from MAA**
**67**	MAA-BED-V205(%)-WNL	**The volume of WNL (%) that receives ≥ 205 Gy of BED calculated from MAA**
**68**	MAA-BED-V400(ml)-TL	**The volume of tumor (ml) that receives ≥ 400 Gy of BED calculated from MAA**
**69**	MAA-BED-V400(ml)-NPL	**The volume of NPL (ml) that receives ≥ 400 Gy of BED calculated from MAA**
**70**	MAA-BED-V400(ml)-WNL	**The volume of WNL (ml) that receives ≥ 400 Gy of BED calculated from MAA**
**71**	MAA-BED-V400(%)-TL	**The volume of tumor (%) that receives ≥ 400 Gy of BED calculated from MAA**
**72**	MAA-BED-V400(%)-NPL	**The volume of NPL (%) that receives ≥ 400 Gy of BED calculated from MAA**
**73**	MAA-BED-V400(%)-WNL	**The volume of WNL (%) that receives ≥ 400 Gy of BED calculated from MAA**
**74**	MAA-Dose-Max (Gy)-TL	**Maximum dose of the tumor from Physical Dose of MAA**
**75**	MAA-Dose-Max (Gy)-NPL	**Maximum dose of the normal perfused liver from Physical Dose of MAA**
**76**	MAA-Dose-Max (Gy)-WNL	**Maximum dose of the whole normal liver from Physical Dose of MAA**
**77**	MAA-Dose-Mean Dose(Gy)-TL	**Mean dose of the tumor from Physical Dose of MAA**
**78**	MAA-Dose-Mean Dose(Gy)-NPL	**Mean dose of the normal perfused liver from Physical Dose of MAA**
**79**	MAA-Dose-Mean Dose(Gy)-WNL	**Mean dose of the whole normal liver from Physical Dose of MAA**
**80**	MAA-Dose-Min (Gy)-TL	**Minimum dose of the Tumor from Physical Dose of MAA**
**81**	MAA-Dose-Min (Gy)-NPL	**Minimum dose of the normal perfused liver from Physical Dose of MAA**
**82**	MAA-Dose-Min (Gy)-WNL	**Minimum dose of the whole normal liver from Physical Dose of MAA**
**83**	MAA-Dose-D50(Gy)-TL	**The Physical Dose from MAA (Gy) received by the 50% of the tumor volume**
**84**	MAA-Dose-D50(Gy)-NPL	**The Physical Dose from MAA (Gy) received by the 50% of the NPL volume**
**85**	MAA-Dose-D50(Gy)-WNL	**The Physical Dose from MAA (Gy) received by the 50% of the WNL volume**
**86**	MAA-Dose-D70(Gy)-TL	**The Physical Dose from MAA (Gy) received by the 70% of the tumor volume**
**87**	MAA-Dose-D70(Gy)-NPL	**The Physical Dose from MAA (Gy) received by the 70% of the NPL volume**
**88**	MAA-Dose-D70(Gy)-WNL	**The Physical Dose from MAA (Gy) received by the 70% of the WNL volume**
**89**	MAA-Dose-D95(Gy)-TL	**The Physical Dose from MAA (Gy) received by the 95% of the tumor volume**
**90**	MAA-Dose-D95(Gy)-NPL	**The Physical Dose from MAA (Gy) received by the 95% of the NPL volume**
**91**	MAA-Dose-D95(Gy)-WNL	**The Physical Dose from MAA (Gy) received by the 95% of the WNL volume**
**92**	MAA-Dose-D98(Gy)-TL	**The Physical Dose from MAA (Gy) received by the 98% of the tumor volume**

**93**	MAA-Dose-D98(Gy)-NPL	**The Physical Dose from MAA (Gy) received by the 98% of the NPL volume**
**94**	MAA-Dose-D98(Gy)-WNL	**The Physical Dose from MAA (Gy) received by the 98% of the WNL volume**
**95**	MAA-Dose-Homogeneity-index-TL	**The ratio of D5/D95 in Tumor from Physical Dose calculated from MAA**
**96**	MAA-Dose-V20(ml)-TL	**The volume of tumor (ml) that receives ≥ 20 Gy of Physical Dose calculated from MAA**
**97**	MAA-Dose-V20(ml)-NPL	**The volume of NPL (ml) that receives ≥ 20 Gy of Physical Dose calculated from MAA**
**98**	MAA-Dose-V20(ml)-WNL	**The volume of WNL (ml) that receives ≥ 20 Gy of Physical Dose calculated from MAA**
**99**	MAA-Dose-V20(%)-TL	**The volume of tumor (m%) that receives ≥ 20 Gy of Physical Dose calculated from MAA**
**100**	MAA-Dose-V20(%)-NPL	**The volume of NPL (%) that receives ≥ 20 Gy of Physical Dose calculated from MAA**
**101**	MAA-Dose-V20(%)-WNL	**The volume of WNL (%) that receives ≥ 20 Gy of Physical Dose calculated from MAA**
**102**	MAA-Dose-V30(ml)-TL	**The volume of tumor (ml) that receives ≥ 30 Gy of Physical Dose calculated from MAA**
**103**	MAA-Dose-V30(ml)-NPL	**The volume of NPL (ml) that receives ≥ 30 Gy of Physical Dose calculated from MAA**
**104**	MAA-Dose-V30(ml)-WNL	**The volume of WNL (ml) that receives ≥ 30 Gy of Physical Dose calculated from MAA**
**105**	MAA-Dose-V30(%)-TL	**The volume of tumor (%) that receives ≥ 30 Gy of Physical Dose calculated from MAA**
**106**	MAA-Dose-V30(%)-NPL	**The volume of NPL (%) that receives ≥ 30 Gy of Physical Dose calculated from MAA**
**107**	MAA-Dose-V30(%)-WNL	**The volume of WNL (%) that receives ≥ 30 Gy of Physical Dose calculated from MAA**
**108**	MAA-Dose-V50(ml)-TL	**The volume of tumor (ml) that receives ≥ 50 Gy of Physical Dose calculated from MAA**
**109**	MAA-Dose-V50(ml)-NPL	**The volume of NPL (ml) that receives ≥ 50 Gy of Physical Dose calculated from MAA**
**110**	MAA-Dose-V50(ml)-WNL	**The volume of WNL (ml) that receives ≥ 50 Gy of Physical Dose calculated from MAA**
**111**	MAA-Dose-V50(%)-TL	**The volume of tumor (%) that receives ≥ 50 Gy of Physical Dose calculated from MAA**
**112**	MAA-Dose-V50(%)-NPL	**The volume of NPL (%) that receives ≥ 50 Gy of Physical Dose calculated from MAA**
**113**	MAA-Dose-V50(%)-WNL	**The volume of WNL (%) that receives ≥ 50 Gy of Physical Dose calculated from MAA**
**114**	MAA-Dose-V70(ml)-TL	**The volume of tumor (ml) that receives ≥ 70 Gy of Physical Dose calculated from MAA**
**115**	MAA-Dose-V70(ml)-NPL	**The volume of NPL (ml) that receives ≥ 70 Gy of Physical Dose calculated from MAA**
**116**	MAA-Dose-V70(ml)-WNL	**The volume of WNL (ml) that receives ≥ 70 Gy of Physical Dose calculated from MAA**
**117**	MAA-Dose-V70(%)-TL	**The volume of tumor (%) that receives ≥ 70 Gy of Physical Dose calculated from MAA**
**118**	MAA-Dose-V70(%)-NPL	**The volume of NPL (%) that receives ≥ 70 Gy of Physical Dose calculated from MAA**
**119**	MAA-Dose-V70(%)-WNL	**The volume of WNL (%) that receives ≥ 70 Gy of Physical Dose calculated from MAA**
**120**	MAA-Dose-V90(ml)-TL	**The volume of tumor (ml) that receives ≥ 90 Gy of Physical Dose calculated from MAA**
**121**	MAA-Dose-V90(ml)-NPL	**The volume of NPL (ml) that receives ≥ 90 Gy of Physical Dose calculated from MAA**
**122**	MAA-Dose-V90(ml)-WNL	**The volume of WNL (ml) that receives ≥ 90 Gy of Physical Dose calculated from MAA**
**123**	MAA-Dose-V90(%)-TL	**The volume of tumor (%) that receives ≥ 90 Gy of Physical Dose calculated from MAA**
**124**	MAA-Dose-V90(%)-NPL	**The volume of NPL (%) that receives ≥ 90 Gy of Physical Dose calculated from MAA**
**125**	MAA-Dose-V90(%)-WNL	**The volume of WNL (%) that receives ≥ 90 Gy of Physical Dose calculated from MAA**
**126**	MAA-Dose-V120(ml)-TL	**The volume of tumor (ml) that receives ≥ 120 Gy of Physical Dose calculated from MAA**
**127**	MAA-Dose-V120(ml)-NPL	**The volume of NPL (ml) that receives ≥ 120 Gy of Physical Dose calculated from MAA**
**128**	MAA-Dose-V120(ml)-WNL	**The volume of WNL (ml) that receives ≥ 120 Gy of Physical Dose calculated from MAA**
**129**	MAA-Dose-V120(%)-TL	**The volume of tumor (%) that receives ≥ 120 Gy of Physical Dose calculated from MAA**
**130**	MAA-Dose-V120(%)-NPL	**The volume of NPL (%) that receives ≥ 120 Gy of Physical Dose calculated from MAA**
**131**	MAA-Dose-V120(%)-WNL	**The volume of WNL (%) that receives ≥ 120 Gy of Physical Dose calculated from MAA**
**132**	MAA-Dose-V205(ml)-TL	**The volume of tumor (ml) that receives ≥ 205 Gy of Physical Dose calculated from MAA**
**133**	MAA-Dose-V205(ml)-NPL	**The volume of NPL (ml) that receives ≥ 205 Gy of Physical Dose calculated from MAA**
**134**	MAA-Dose-V205(ml)-WNL	**The volume of WNL (ml) that receives ≥ 205 Gy of Physical Dose calculated from MAA**
**135**	MAA-Dose-V205(%)-TL	**The volume of tumor (%) that receives ≥ 205 Gy of Physical Dose calculated from MAA**
**136**	MAA-Dose-V205(%)-NPL	**The volume of NPL (%) that receives ≥ 205 Gy of Physical Dose calculated from MAA**
**137**	MAA-Dose-V205(%)-WNL	**The volume of WNL (%) that receives ≥ 205 Gy of Physical Dose calculated from MAA**
**138**	MAA-Dose-V400(ml)-TL	**The volume of tumor (ml) that receives ≥ 400 Gy of Physical Dose calculated from MAA**
**139**	MAA-Dose-V400(ml)-NPL	**The volume of NPL (ml) that receives ≥ 400 Gy of Physical Dose calculated from MAA**
**140**	MAA-Dose-V400(ml)-WNL	**The volume of WNL (ml) that receives ≥ 400 Gy of Physical Dose calculated from MAA**

**141**	MAA-Dose-V400(%)-TL	**The volume of tumor (%) that receives ≥ 400 Gy of Physical Dose calculated from MAA**
**142**	MAA-Dose-V400(%)-NPL	**The volume of NPL (%) that receives ≥ 400 Gy of Physical Dose calculated from MAA**
**143**	MAA-Dose-V400(%)-WNL	**The volume of WNL (%) that receives ≥ 400 Gy of Physical Dose calculated from MAA**
**144**	MAA-TNR-NPL	**TNR calculated for MAA with mass of NPL**
**145**	MAA-TNR-WNL	**TNR calculated for MAA with mass of WNL**
**146**	LSF	**Lung shunt fraction (%)**
**147**	Y90-BED-Max (Gy)-TL	**Maximum dose of the tumor from BED of Y90**
**148**	Y90-BED-Max (Gy)-NPL	**Maximum dose of the normal perfused liver from BED of Y90**
**149**	Y90-BED-Max (Gy)-WNL	**Maximum dose of the whole normal liver from BED of Y90**
**150**	Y90-BED-Mean Dose (Gy)-TL	**Mean dose of the tumor from BED of Y90**
**151**	Y90-BED-Mean Dose (Gy)-NPL	**Mean dose of the normal perfused liver from BED of Y90**
**152**	Y90-BED-Mean Dose (Gy)-WNL	**Mean dose of the whole normal liver from BED of Y90**
**153**	Y90-BED-Min (Gy)-TL	**Minimum dose of the Tumor from BED of Y90**
**154**	Y90-BED-Min (Gy)-NPL	**Minimum dose of the normal perfused liver from BED of Y90**
**155**	Y90-BED-Min (Gy)-WNL	**Minimum dose of the whole normal liver from BED of Y90**
**156**	Y90-BED-D50(Gy)-TL	**The biologically effective dose from Y90(Gy) received by the 50% of the tumor volume**
**157**	Y90-BED-D50(Gy)-NPL	**The biologically effective dose from Y90 (Gy) received by the 50% of the NPL volume**
**158**	Y90-BED-D50(Gy)-WNL	**The biologically effective dose from Y90 (Gy) received by the 50% of the WNL volume**
**159**	Y90-BED-D70(Gy)-TL	**The biologically effective dose from Y90 (Gy) received by the 70% of the tumor volume**
**160**	Y90-BED-D70(Gy)-NPL	**The biologically effective dose from Y90 (Gy) received by the 70% of the NPL volume**
**161**	Y90-BED-D70(Gy)-WNL	**The biologically effective dose from Y90(Gy) received by the 70% of the WNL volume**
**162**	Y90-BED-D95(Gy)-TL	**The biologically effective dose from Y90 (Gy) received by the 95% of the tumor volume**
**163**	Y90-BED-D95(Gy)-NPL	**The biologically effective dose from Y90 (Gy) received by the 95% of the NPL volume**
**164**	Y90-BED-D95(Gy)-WNL	**The biologically effective dose from Y90 (Gy) received by the 95% of the WNL volume**
**165**	Y90-BED-D98(Gy)-TL	**The biologically effective dose from Y90 (Gy) received by the 98% of the tumor volume**
**166**	Y90-BED-D98(Gy)-NPL	**The biologically effective dose from Y90 (Gy) received by the 98% of the NPL volume**
**167**	Y90-BED-D98(Gy)-WNL	**The biologically effective dose from Y90 (Gy) received by the 98% of the WNL volume**
**168**	Y90-BED-Hemogenity-index-TL	**The ratio of D5/D95 in Tumor from biologically effective dose calculated from Y90**
**169**	Y90-BED-V20(ml)-TL	**The volume of tumor (ml) that receives ≥ 20 Gy of BED calculated from Y90**
**170**	Y90-BED-V20(ml)-NPL	**The volume of NPL (ml) that receives ≥ 20 Gy of BED calculated from Y90**
**171**	Y90-BED-V20(ml)-WNL	**The volume of WNL (ml) that receives ≥ 20 Gy of BED calculated from Y90**
**172**	Y90-BED-V20(%)-TL	**The volume of tumor (m%) that receives ≥ 20 Gy of BED calculated from Y90**
**173**	Y90-BED-V20(%)-NPL	**The volume of NPL (%) that receives ≥ 20 Gy of BED calculated from Y90**
**174**	Y90-BED-V20(%)-WNL	**The volume of WNL (%) that receives ≥ 20 Gy of BED calculated from Y90**
**175**	Y90-BED-V30(ml)-TL	**The volume of tumor (ml) that receives ≥ 30 Gy of BED calculated from Y90**
**176**	Y90-BED-V30(ml)-NPL	**The volume of NPL (ml) that receives ≥ 30 Gy of BED calculated from Y90**
**177**	Y90-BED-V30(ml)-WNL	**The volume of WNL (ml) that receives ≥ 30 Gy of BED calculated from Y90**
**178**	Y90-BED-V30(%)-TL	**The volume of tumor (%) that receives ≥ 30 Gy of BED calculated from Y90**
**179**	Y90-BED-V30(%)-NPL	**The volume of NPL (%) that receives ≥ 30 Gy of BED calculated from Y90**
**180**	Y90-BED-V30(%)-WNL	**The volume of WNL (%) that receives ≥ 30 Gy of BED calculated from Y90**
**181**	Y90-BED-V50(ml)-TL	**The volume of tumor (ml) that receives ≥ 50 Gy of BED calculated from Y90**
**182**	Y90-BED-V50(ml)-NPL	**The volume of NPL (ml) that receives ≥ 50 Gy of BED calculated from Y90**
**183**	Y90-BED-V50(ml)-WNL	**The volume of WNL (ml) that receives ≥ 50 Gy of BED calculated from Y90**
**184**	Y90-BED-V50(%)-TL	**The volume of tumor (%) that receives ≥ 50 Gy of BED calculated from Y90**
**185**	Y90-BED-V50(%)-NPL	**The volume of NPL (%) that receives ≥ 50 Gy of BED calculated from Y90**
**186**	Y90-BED-V50(%)-WNL	**The volume of WNL (%) that receives ≥ 50 Gy of BED calculated from Y90**
**187**	Y90-BED-V70(ml)-TL	**The volume of tumor (ml) that receives ≥ 70 Gy of BED calculated from Y90**
**188**	Y90-BED-V70(ml)-NPL	**The volume of NPL (ml) that receives ≥ 70 Gy of BED calculated from Y90**

189	Y90-BED-V70(ml)-WNL	The volume of WNL (ml) that receives ≥ 70 Gy of BED calculated from Y90
190	Y90-BED-V70(%)-TL	The volume of tumor (%) that receives ≥ 70 Gy of BED calculated from Y90
191	Y90-BED-V70(%)-NPL	The volume of NPL (%) that receives ≥ 70 Gy of BED calculated from Y90
192	Y90-BED-V70(%)-WNL	The volume of WNL (%) that receives ≥ 70 Gy of BED calculated from Y90
193	Y90-BED-V90(ml)-TL	The volume of tumor (ml) that receives ≥ 90 Gy of BED calculated from Y90
194	Y90-BED-V90(ml)-NPL	The volume of NPL (ml) that receives ≥ 90 Gy of BED calculated from Y90
195	Y90-BED-V90(ml)-WNL	The volume of WNL (ml) that receives ≥ 90 Gy of BED calculated from Y90
196	Y90-BED-V90(%)-TL	The volume of tumor (%) that receives ≥ 90 Gy of BED calculated from Y90
197	Y90-BED-V90(%)-NPL	The volume of NPL (%) that receives ≥ 90 Gy of BED calculated from Y90
198	Y90-BED-V90(%)-WNL	The volume of WNL (%) that receives ≥ 90 Gy of BED calculated from Y90
199	Y90-BED-V120(ml)-TL	The volume of tumor (ml) that receives ≥ 120 Gy of BED calculated from Y90
200	Y90-BED-V120(ml)-NPL	The volume of NPL (ml) that receives ≥ 120 Gy of BED calculated from Y90
201	Y90-BED-V120(ml)-WNL	The volume of WNL (ml) that receives ≥ 120 Gy of BED calculated from Y90
202	Y90-BED-V120(%)-TL	The volume of tumor (%) that receives ≥ 120 Gy of BED calculated from Y90
203	Y90-BED-V120(%)-NPL	The volume of NPL (%) that receives ≥ 120 Gy of BED calculated from Y90
204	Y90-BED-V120(%)-WNL	The volume of WNL (%) that receives ≥ 120 Gy of BED calculated from Y90
205	Y90-BED-V205(ml)-TL	The volume of tumor (ml) that receives ≥ 205 Gy of BED calculated from Y90
206	Y90-BED-V205(ml)-NPL	The volume of NPL (ml) that receives ≥ 205 Gy of BED calculated from Y90
207	Y90-BED-V205(ml)-WNL	The volume of WNL (ml) that receives ≥ 205 Gy of BED calculated from Y90
208	Y90-BED-V205(%)-TL	The volume of tumor (%) that receives ≥ 205 Gy of BED calculated from Y90
209	Y90-BED-V205(%)-NPL	The volume of NPL (%) that receives ≥ 205 Gy of BED calculated from Y90
210	Y90-BED-V205(%)-WNL	The volume of WNL (%) that receives ≥ 205 Gy of BED calculated from Y90
211	Y90-BED-V400(ml)-TL	The volume of tumor (ml) that receives ≥ 400 Gy of BED calculated from Y90
212	Y90-BED-V400(ml)-NPL	The volume of NPL (ml) that receives ≥ 400 Gy of BED calculated from Y90
213	Y90-BED-V400(ml)-WNL	The volume of WNL (ml) that receives ≥ 400 Gy of BED calculated from Y90
214	Y90-BED-V400(%)-TL	The volume of tumor (%) that receives ≥ 400 Gy of BED calculated from Y90
215	Y90-BED-V400(%)-NPL	The volume of NPL (%) that receives ≥ 400 Gy of BED calculated from Y90
216	Y90-BED-V400(%)-WNL	The volume of WNL (%) that receives ≥ 400 Gy of BED calculated from Y90
217	Y90-Dose-Max (Gy)-TL	Maximum dose of the tumor from Physical Dose of Y90
218	Y90-Dose-Max (Gy)-NPL	Maximum dose of the normal perfused liver from Physical Dose of Y90
219	Y90-Dose-Max (Gy)-WNL	Maximum dose of the whole normal liver from Physical Dose of Y90
220	Y90-Dose-Mean Dose (Gy)-TL	Mean dose of the tumor from Physical Dose of Y90
221	Y90-Dose-Mean Dose (Gy)-NPL	Mean dose of the normal perfused liver from Physical Dose of Y90
222	Y90-Dose-Mean Dose (Gy)-WNL	Mean dose of the whole normal liver from Physical Dose of Y90
223	Y90-Dose-Min (Gy)-TL	Minimum dose of the Tumor from Physical Dose of Y90
224	Y90-Dose-Min (Gy)-NPL	Minimum dose of the normal perfused liver from Physical Dose of Y90
225	Y90-Dose-Min (Gy)-WNL	Minimum dose of the whole normal liver from Physical Dose of Y90
226	Y90-Dose-D50(Gy)-TL	The Physical Dose from Y90 (Gy) received by the 50% of the tumor volume
227	Y90-Dose-D50(Gy)-NPL	The Physical Dose from Y90 (Gy) received by the 50% of the NPL volume
228	Y90-Dose-D50(Gy)-WNL	The Physical Dose from Y90 (Gy) received by the 50% of the WNL volume
229	Y90-Dose-D70(Gy)-TL	The Physical Dose from Y90 (Gy) received by the 70% of the tumor volume
230	Y90-Dose-D70(Gy)-NPL	The Physical Dose from Y90 (Gy) received by the 70% of the NPL volume
231	Y90-Dose-D70(Gy)-WNL	The Physical Dose from Y90(Gy) received by the 70% of the WNL volume
232	Y90-Dose-D95(Gy)-TL	The Physical Dose from Y90 (Gy) received by the 95% of the tumor volume
233	Y90-Dose-D95(Gy)-NPL	The Physical Dose from Y90(Gy) received by the 95% of the NPL volume
234	Y90-Dose-D95(Gy)-WNL	The Physical Dose from Y90 (Gy) received by the 95% of the WNL volume
235	Y90-Dose-D98(Gy)-TL	The Physical Dose from Y90 (Gy) received by the 98% of the tumor volume
236	Y90-Dose-D98(Gy)-NPL	The Physical Dose from Y90 (Gy) received by the 98% of the NPL volume

**237**	Y90-Dose-D98(Gy)-WNL	**The Physical Dose from Y90(Gy) received by the 98% of the WNL volume**
**238**	Y90-Dose-Hemogenity-index-TL	**The ratio of D5/D95 in Tumor from Physical Dose calculated from Y90**
**239**	Y90-Dose-V20(ml)-TL	**The volume of tumor (ml) that receives ≥ 20 Gy of Physical Dose calculated from Y90**
**240**	Y90-Dose-V20(ml)-NPL	**The volume of NPL (ml) that receives ≥ 20 Gy of Physical Dose calculated from Y90**
**241**	Y90-Dose-V20(ml)-WNL	**The volume of WNL (ml) that receives ≥ 20 Gy of Physical Dose calculated from Y90**
**242**	Y90-Dose-V20(%)-TL	**The volume of tumor (m%) that receives ≥ 20 Gy of Physical Dose calculated from Y90**
**243**	Y90-Dose-V20(%)-NPL	**The volume of NPL (%) that receives ≥ 20 Gy of Physical Dose calculated from Y90**
**244**	Y90-Dose-V20(%)-WNL	**The volume of WNL (%) that receives ≥ 20 Gy of Physical Dose calculated from Y90**
**245**	Y90-Dose-V30(ml)-TL	**The volume of tumor (ml) that receives ≥ 30 Gy of Physical Dose calculated from Y90**
**246**	Y90-Dose-V30(ml)-NPL	**The volume of NPL (ml) that receives ≥ 30 Gy of Physical Dose calculated from Y90**
**247**	Y90-Dose-V30(ml)-WNL	**The volume of WNL (ml) that receives ≥ 30 Gy of Physical Dose calculated from Y90**
**248**	Y90-Dose-V30(%)-TL	**The volume of tumor (%) that receives ≥ 30 Gy of Physical Dose calculated from Y90**
**249**	Y90-Dose-V30(%)-NPL	**The volume of NPL (%) that receives ≥ 30 Gy of Physical Dose calculated from Y90**
**250**	Y90-Dose-V30(%)-WNL	**The volume of WNL (%) that receives ≥ 30 Gy of Physical Dose calculated from Y90**
**251**	Y90-Dose-V50(ml)-TL	**The volume of tumor (ml) that receives ≥ 50 Gy of Physical Dose calculated from Y90**
**252**	Y90-Dose-V50(ml)-NPL	**The volume of NPL (ml) that receives ≥ 50 Gy of Physical Dose calculated from Y90**
**253**	Y90-Dose-V50(ml)-WNL	**The volume of WNL (ml) that receives ≥ 50 Gy of Physical Dose calculated from Y90**
**254**	Y90-Dose-V50(%)-TL	**The volume of tumor (%) that receives ≥ 50 Gy of Physical Dose calculated from Y90**
**255**	Y90-Dose-V50(%)-NPL	**The volume of NPL (%) that receives ≥ 50 Gy of Physical Dose calculated from Y90**
**256**	Y90-Dose-V50(%)-WNL	**The volume of WNL (%) that receives ≥ 50 Gy of Physical Dose calculated from Y90**
**257**	Y90-Dose-V70(ml)-TL	**The volume of tumor (ml) that receives ≥ 70 Gy of Physical Dose calculated from Y90**
**258**	Y90-Dose-V70(ml)-NPL	**The volume of NPL (ml) that receives ≥ 70 Gy of Physical Dose calculated from Y90**
**259**	Y90-Dose-V70(ml)-WNL	**The volume of WNL (ml) that receives ≥ 70 Gy of Physical Dose calculated from Y90**
**260**	Y90-Dose-V70(%)-TL	**The volume of tumor (%) that receives ≥ 70 Gy of Physical Dose calculated from Y90**
**261**	Y90-Dose-V70(%)-NPL	**The volume of NPL (%) that receives ≥ 70 Gy of Physical Dose calculated from Y90**
**262**	Y90-Dose-V70(%)-WNL	**The volume of WNL (%) that receives ≥ 70 Gy of Physical Dose calculated from Y90**
**263**	Y90-Dose-V90(ml)-TL	**The volume of tumor (ml) that receives ≥ 90 Gy of Physical Dose calculated from Y90**
**264**	Y90-Dose-V90(ml)-NPL	**The volume of NPL (ml) that receives ≥ 90 Gy of Physical Dose calculated from Y90**
**265**	Y90-Dose-V90(ml)-WNL	**The volume of WNL (ml) that receives ≥ 90 Gy of Physical Dose calculated from Y90**
**266**	Y90-Dose-V90(%)-TL	**The volume of tumor (%) that receives ≥ 90 Gy of Physical Dose calculated from Y90**
**267**	Y90-Dose-V90(%)-NPL	**The volume of NPL (%) that receives ≥ 90 Gy of Physical Dose calculated from Y90**
**268**	Y90-Dose-V90(%)-WNL	**The volume of WNL (%) that receives ≥ 90 Gy of Physical Dose calculated from Y90**
**269**	Y90-Dose-V120(ml)-TL	**The volume of tumor (ml) that receives ≥ 120 Gy of Physical Dose calculated from Y90**
**270**	Y90-Dose-V120(ml)-NPL	**The volume of NPL (ml) that receives ≥ 120 Gy of Physical Dose calculated from Y90**
**271**	Y90-Dose-V120(ml)-WNL	**The volume of WNL (ml) that receives ≥ 120 Gy of Physical Dose calculated from Y90**
**272**	Y90-Dose-V120(%)-TL	**The volume of tumor (%) that receives ≥ 120 Gy of Physical Dose calculated from Y90**
**273**	Y90-Dose-V120(%)-NPL	**The volume of NPL (%) that receives ≥ 120 Gy of Physical Dose calculated from Y90**
**274**	Y90-Dose-V120(%)-WNL	**The volume of WNL (%) that receives ≥ 120 Gy of Physical Dose calculated from Y90**
**275**	Y90-Dose-V205(ml)-TL	**The volume of tumor (ml) that receives ≥ 205 Gy of Physical Dose calculated from Y90**
**276**	Y90-Dose-V205(ml)-NPL	**The volume of NPL (ml) that receives ≥ 205 Gy of Physical Dose calculated from Y90**
**277**	Y90-Dose-V205(ml)-WNL	**The volume of WNL (ml) that receives ≥ 205 Gy of Physical Dose calculated from Y90**
**278**	Y90-Dose-V205(%)-TL	**The volume of tumor (%) that receives ≥ 205 Gy of Physical Dose calculated from Y90**
**279**	Y90-Dose-V205(%)-NPL	**The volume of NPL (%) that receives ≥ 205 Gy of Physical Dose calculated from Y90**
**280**	Y90-Dose-V205(%)-WNL	**The volume of WNL (%) that receives ≥ 205 Gy of Physical Dose calculated from Y90**
**281**	Y90-Dose-V400(ml)-TL	**The volume of tumor (ml) that receives ≥ 400 Gy of Physical Dose calculated from Y90**
**282**	Y90-Dose-V400(ml)-NPL	**The volume of NPL (ml) that receives ≥ 400 Gy of Physical Dose calculated from Y90**
**283**	Y90-Dose-V400(ml)-WNL	**The volume of WNL (ml) that receives ≥ 400 Gy of Physical Dose calculated from Y90**
**284**	Y90-Dose-V400(%)-TL	**The volume of tumor (%) that receives ≥ 400 Gy of Physical Dose calculated from Y90**

**285**	Y90-Dose-V400(%)-NPL	**The volume of NPL (%) that receives ≥ 400 Gy of Physical Dose calculated from Y90**
**286**	Y90-Dose-V400(%)-WNL	**The volume of WNL (%) that receives ≥ 400 Gy of Physical Dose calculated from Y90**
**287**	Y90-TNR-NPL	**TNR calculated for Y90 with mass of NPL**
**288**	Y90-TNR-WNL	**TNR calculated for Y90 with mass of WNL**
**289**	Injected-Activity-Y90	**Injected activity of Y90 to the patient**
**290**	Age	**-**
**291**	Aim of RE	**Weather it is right or left Lobectomy, Palliative treatment, or Segmentectomy**
**292**	Previous EBRT or other treatments	**Whether the patiend had no previous treatment or had Chemoembolization, Chemoembolization+ Luthathera, Chemoembolization + sorafenib, Surgery, Chemotherapy, Previous SIRT (4 years ago), hormone therapy**
**293**	PVT	**Portal vein thrombosis**
**294**	Hepatitis	**-**
**295**	Ascites	-
**296**	Cirrhosis	-
**297**	AFP	**Alphafeto protein level in blood (μg/L)**
**298**	Albumin	**-**
**299**	Bilirubin	-
**300**	AST	**Aspartate aminotransferase(U/L)**
**301**	ALT	**Alanine aminotransferase(U/L)**
**302**	Hb	**Hemoglobin (g/L)**
**303**	WBC	**Leucocyte count (×10^9/L)**
**304**	Platelet	**(×10^9/L)**
**305**	ECOG	**Performance based on eastern cooperative oncology group**

Table 2. The median ± SD of the dosimetry parameters for tumor and OARs (NPL and WNL). * HI: homogeneity index.

Anatomical Structure	Metric	^{99m}TC-MAA-Dose	^{90}Y-Dose	^{99m}TC-MAA- BED	^{90}Y- BED
TL	Max (Gy)	1027.08 ± 781.67	605.38 ± 920.78	3331.86 ± 7768.92	1442.11± 9753.4
	Mean (Gy)	345.6 ± 179.6	256.23 ± 143.66	662.95 ± 521.65	413.78 ± 583.73
	Min (Gy)	36.01 ± 29.11	52.33 ± 34.09	38.98 ± 34.85	58.6 ± 44.38
	D50(Gy)	310.39 ± 151.5	242.3 ± 103.9	530.30 ± 333.09	376.31± 232.73
	D70 (Gy)	230.93 ± 98.62	178.55 ± 85.3	352.35 ± 172.68	251.34 ±162.44
	D95(Gy)	131.64 ± 63.06	103.4 ± 61.15	171.2 ± 94.12	127.78 ± 96.8
	D98(Gy)	93.02 ± 55.1	87.77 ± 53.5	112.74 ±7 9.94	105.36 ± 80.13
	HI*	606.29 ± 1205.16	507.67 ± 380.9	1064.25 ±2741.92	872.88 ± 1187.2
	V20(ml)	153.9 ± 215.22	229.42 ± 203.48	153.9 ± 218.15	229.42 ± 203.69
	V20(%)	100 ± 6.25	100± 0.22	100 ± 5.95	100 ± 0.16
	V30 (ml)	153.35 ± 144.25	229.42 ± 199.84	153.79 ± 188.53	229.42 ± 200.39
	V30 (%)	100 ± 9.5	100 ± 1.15	100 ± 8.95	100 ± 1.03
	V50(ml)	154.34 ± 169.37	229.42 ± 188.17	153.46 ±150.37	229.42 ± 192
	V50(%)	99.84 ± 14.16	100 ± 5.3	99.89 ± 13.33	100 ± 3.05
	V70(ml)	151.25 ± 128.15	204.5 ± 165.19	152.03±133.5	221.92 ± 176.3
	V70(%)	99.21 ± 16.3	99.53 ± 11.76	99.34± 15.6	99.77 ± 9.28
	V90(ml)	146.95 ± 118.95	204.5 ± 139.87	149.82 ± 124.58	204.5 ± 157.1
	V90(%)	98.26 ± 17.33	97.72 ± 16.05	98.89±16.7	99.17 ± 13.37
	V120(ml)	141 ± 107.6	199.32 ± 108.43	145.3±116.07	203.95 ± 130.13
	V120(%)	96.11 ± 18.45	89.94 ± 19.48	97.84 ±17.6	96.28 ± 17.07
	V205(ml)	134.4 ± 82.26	122.04 ± 73.98	140.45 ± 12.3	155.66 ± 86.1
	V205(%)	78.27 ± 22.14	57.74 ± 27.32	91.86 ± 19.8	80.37 ± 22.63
	V400(ml)	60.85 ± 54.4	23.04 ± 44.3	113.77 ±75.11	93.37 ± 64.41
	V400(%)	26.8 ± 22.57	12.08 ± 19.73	66.76 ± 23.41	45.66 ± 27.18
NPL	Max (Gy)	510.8 ± 365.17	295.76± 289.44	1445.44 ± 2768.77	536.26 ± 1964.92
	Mean (Gy)	49.5 ± 73.5	65.86 ± 36.52	65.6 ± 188.96	104.66 ± 62.5
	Min (Gy)	0.7 ± 7.8	7.21±7.26	0 ± .01	0
	D50(Gy)	27.86 ± 64.6	44.95 ±34.94	32.18 ± 127.42	54.33 ± 55.73
	D70 (Gy)	12.3 ± 46.7	27.22 ± 27.5	12.9 ± 82.3	30.91 ± 41.16
	D95(Gy)	3.7 ± 19.7	14.5 ± 15.26	4.6 ± 29.67	15.6±20.62
	D98(Gy)	3 ± 14.7	12.23 ± 11.75	3.06 ± 21.6	13.31 ± 16.84
	V20(ml)	447.9 ± 442.1	755.3± 436.4	417.72 ± 405.1	769.85 ± 414.76
	V20(%)	61.7 ± 97.67	83.1 ± 16.63	54.64 ± 22.86	81.66 ± 13.81
	V30 (ml)	350.25 ± 403.5	468.76 ± 419.14	389.17± 380.2	507.57 ± 398.83
	V30 (%)	46.6 ± 94.4	66.58 ± 25.06	47.85 ± 25.35	65.72± 20.94
	V50(ml)	249.37 ± 358.44	311.44 ± 385.21	302.07 ± 349.41	315.63± 380.73
	V50(%)	33.28 ±87.4	45.14 ± 29.68	34.23 ± 27.19	49.21± 27.13
	V70(ml)	208.58 ± 317.26	234.05 ± 347.06	266.68 ± 323.83	245.74 ± 356.15
	V70(%)	24.7 ± 79.7	31.47 ± 28.06	27.25 ± 27.11	36.84 ± 27.74
	V90(ml)	174.5 ± 284.5	196.45 ± 297.16	216.96 ± 300.102	202.85 ± 332.64
	V90(%)	17.62 ± 70.91	22.96 ± 24.6	22.13 ± 26.38	29.28 ± 26.53
	V120(ml)	103.19 ± 227.1	89.85 ± 190.94	171 ± 273.45	171.65 ± 290.96
	V120(%)	11.04 ± 55.3	13.85 ± 17.61	17.04 ± 25.09	21.91 ± 23.7
	V205(ml)	44.09 ± 60.2	6.39 ± 40.1	98.9± 186.83	72.21 ±131.68
	V205(%)	4.48 ± 31.9	2.26 ± 6.05	8.08 ± 20.86	9.96 ± 13.66
	V400(ml)	1.54 ± 13.97	0 ± 8.62	39.02 ± 49.77	4.63± 32.57
	V400(%)	0.15 ± 6.05	0 ± 0.67	4.01± 12.82	1.08 ± 4.88
	TNR-NPL	40.11 ± 85.31	15.94 ± 26.64	-	-
WNL	Max (Gy)	587.42 ± 364.55	267.68 ± 293.25	1488.72 ± 2750.48	536.26 ± 1964.92
	Mean (Gy)	37.84 ± 23.08	41.6 ± 23.24	52.23 ± 45.7	62.08 ± 39.52
	Min (Gy)	0.17 ± 0.21	2.76 ± 1.75	0.17 ± 0.21	2.8 ± 1.8
	D50(Gy)	6.46 ± 23.04	24.17 ± 22.41	6.62 ± 31.78	26.35 ± 30.83
	D70 (Gy)	3.72 ± 9.5	15.35 ± 13.87	3.76 ± 10.97	16.2 ± 17.05
	D95(Gy)	1.36 ± 1.04	7.19 ± 5.48	1.36 ± 1.07	7.38 ± 5.93
	D98(Gy)	0.96 ± 0.75	5.8 ± 4.23	0.95 ± 0.74	5.91 ± 4.46
	V20(ml)	428.97 ± 399.4	900.49 ± 447.84	435.14 ± 403.22	958.15 ± 445.03
	V20(%)	30.42 ± 97.47	57.73 ± 21.92	32.44 ± 18.4	61.71 ± 21.78
	V30 (ml)	365.57 ± 391.75	572.29 ± 452.82	405.15 ± 384.77	616.72± 455.08
	V30 (%)	24.78 ± 93.35	39.12 ± 22	25.57 ± 17.2	45.35 ± 22.17
	V50(ml)	300.97 ± 351.95	367.23 ± 5.3	332.5 ±356.9	425.99± 424.98
	V50(%)	19.84 ± 85.57	24.97 ± 19.45	22.12 ± 15.6	28.06 ± 20.35
	V70(ml)	223.13 ± 312.67	250.03 ± 359.88	283.55 ± 330.71	310.67 ± 388.33
	V70(%)	17.07 ± 77.5	18.26 ± 16.9	18.14 ± 14.46	20.9 ±18.44
	V90(ml)	170 ± 280.3	197.34 ± 303.52	25467 ± 306.41	245.18 ± 356.9
	V90(%)	14.75 ± 68.33	14.29 ± 14.16	16.97 ± 13.5	18.04 ± 16.74
	V120(ml)	108.15 ± 224.5	102.86 ± 190.26	175.73 ± 277.91	198 ± 304.08
	V120 (%)	8.67 ± 52.5	8.64 ± 9.31	14.81 ± 12.31	14.3 ± 14.19
	V205(ml)	42.33 ± 61.42	8.26 ± 40.17	98.9 ± 19.8	81.47 ± 133.25
	V205(%)	2.5 ± 29.07	0.49 ± 2.77	6.75 ± 8.5	7.44 ± 7.08
	V400(ml)	154 ± 16.03	0± 8.81	39.68 ± 53.75	6.4 ± 33.77
	V400(%)	0.09 ± 3.27	0± 0.53	1.9 ± 3.5	0.37 ± 2.28
	TNR-WNL	79.41 ± 132.16	45.71 ± 48.74	-	-

Table 3. the most effective univariate predictors of "OS" highlighted as statistically significant (p-value < 0.05) variables. The Hazard Ratio (HR) is provided within the 95% confidence interval (CI), along with the Wald test statistic, concordance index, and standard error (SE). The cut-off value, set at the median of the feature, delineates the stratification into two distinct groups. HR> 1 indicates an increased risk of the event (OS).

	FEATURE	BETA	HR (95% CI)	WALD-TEST	P-VALUE	CONCORDANCE	SE	CUT OFF
CLINICAL	Ascites	2.2	9.2 (1.8-47)	7.1	0.0077	0.72	0.087	-
	Aim-of-RE	-2.7	0.066 (0.0057-0.78)	4.7	0.0308	0.7	0.078	-
	AST	-2.3	0.1 (0.012-0.86)	4.4	0.0356	0.76	0.06	54
^{99M}TC-MAA	Dose-V205(%)-TL	2.1	8.5 (1-72)	3.9	0.0487	0.7	0.11	78

Table 4. The most effective univariate predictors of "PFS" highlighted as statistically significant (p-value < 0.05) variables. The Hazard Ratio (HR) is provided within the 95% confidence interval (CI), along with the Wald test statistic, concordance index, and standard error (SE). The cut-off value, set at the median of the feature, delineates the stratification into two distinct groups. HR> 1 indicates an increased risk of the event (PFS). [For instance, the feature "Dose-V205(%)-TL" has been stratified based on its median value (78%), the HR of 5.1 means that the group with Dose-V205(%)-TL > 78% is high risk compared to the group with Dose-V205(%)-TL < 78%.

	Feature (Dose map- DVC- structure)	*Beta*	*HR (95% CI)*	*Wald-Test*	*p-Value*	*Concordance*	*SE*	*Cut off*
^{99m}TC-MAA	Dose-V205(%)-TL	1.6	5.1 (1-26)	3.8	0.0496	0.68	0.082	78
	Dose-MAX-NPL-Gy	2	7.3 (1.5-37)	5.9	0.0148	0.73	0.091	510
	Dose-Mean-NPL-Gy	2.1	8.4 (1-70)	3.9	0.0493	0.7	0.086	50
	Dose-D50-NPL-Gy	2.1	8.4 (1-70)	3.9	0.0493	0.7	0.086	28
	Dose-V20(%)-NPL	2.1	8.4 (1-70)	3.9	0.0493	0.7	0.086	62
	Dose-V30(%)-NPL	2.1	8.4 (1-70)	3.9	0.0493	0.7	0.086	47
	Dose-V90(%)-NPL	2.1	8.4 (1-70)	3.9	0.0493	0.7	0.086	18
	Dose-V120(%)-NPL	2.1	8.4 (1-70)	3.9	0.0493	0.7	0.086	11
	Dose-V205(%)-NPL	2.4	11 (1.3-92)	4.7	0.0301	0.72	0.087	4.5
	Dose-V400(ml)-NPL	1.8	6.2 (1.3-31)	5	0.0251	0.71	0.09	1.5
	Dose-V400(%)-NPL	1.8	6.2 (1.3-31)	5	0.0251	0.71	0.09	0.15
	Dose-V120(%)-WNL	2.1	8.4 (1-70)	3.9	0.0493	0.7	0.086	8.7
	Dose-V205(%)-WNL	2.6	13 (1.5-120)	5.3	0.0217	0.73	0.087	2.5
	Dose-V400(ml)-WNL	1.8	6.2 (1.3-31)	5	0.0251	0.71	0.09	1.5
	Dose-V400(%)-WNL	1.8	6.2 (1.3-31)	5	0.0251	0.71	0.09	0.092
	BED-Mean-NPL-Gy	2.1	8.4 (1-70)	3.9	0.0493	0.7	0.086	66
	BED-D50-NPL-Gy	2.1	8.4 (1-70)	3.9	0.0493	0.7	0.086	32
	BED-V20(%)-NPL	2.1	8.4 (1-70)	3.9	0.0493	0.7	0.086	55
	BED-V30(%)-NPL	2.1	8.4 (1-70)	3.9	0.0493	0.7	0.086	48
	BED-V120(ml)-NPL	2.2	9.1 (1.1-75)	4.1	0.0418	0.73	0.072	170
	BED-V205(%)-NPL	2.4	11 (1.3-92)	4.7	0.0301	0.72	0.087	8.1
	BED-V400(%)-NPL	2.4	11 (1.3-92)	4.7	0.0301	0.72	0.087	4
	BED-V70(ml)-WNL	2.2	9.1 (1.1-75)	4.1	0.0418	0.73	0.072	280
	BED-V90(ml)-WNL	2.2	9.1 (1.1-75)	4.1	0.0418	0.73	0.072	250
	BED-V120(ml)-WNL	2.2	9.1 (1.1-75)	4.1	0.0418	0.73	0.072	180
	BED-V205(%)-WNL	2.4	11 (1.3-92)	4.7	0.0301	0.72	0.087	6.8
	BED-V400(%)-WNL	2.6	13 (1.5-120)	5.3	0.0217	0.73	0.087	1.9
^{90}Y	Dose-Mean-TL-Gy	2.7	15 (1.8-120)	6.2	0.0126	0.78	0.058	260
	Dose-D50-TL-Gy	2.7	15 (1.8-120)	6.2	0.0126	0.78	0.058	240
	Dose-D70-TL-Gy	1.8	6.2 (1.3-30)	5.2	0.0233	0.76	0.066	180
	Dose-V205(%)-TL	2.7	15 (1.8-120)	6.2	0.0126	0.78	0.058	58
	Dose-Mean-NPL-Gy	2.4	11 (1.3-92)	4.7	0.0301	0.72	0.087	66
	Dose-V90(%)-NPL	2.5	12 (1.4-98)	5.2	0.0228	0.73	0.09	23
	Dose-V120(%)-NPL	2.5	12 (1.4-98)	5.2	0.0228	0.73	0.09	14
	Dose-V205(ml) NPL	2.4	11 (1.3-92)	4.7	0.0301	0.72	0.087	6.4
	Dose-V205(%)-NPL	2.4	11 (1.3-92)	4.7	0.0301	0.72	0.087	2.3
	Dose-MAX-WNL-Gy	2.4	11 (1.3-92)	4.7	0.0301	0.72	0.087	270
	Dose-V205(ml)-WNL	2.4	11 (1.3-92)	4.7	0.0301	0.72	0.087	8.3
	Dose-V205(%)-WNL	2.4	11 (1.3-92)	4.7	0.0301	0.72	0.087	0.49
	BED-Mean-TL-Gy	1.9	6.7 (1.4-33)	5.5	0.0187	0.74	0.066	410
	BED-D50-TL-Gy	2.7	15 (1.8-120)	6.2	0.0126	0.78	0.058	380
	BED-D70-TL-Gy	1.8	6.2 (1.3-30)	5.2	0.0233	0.76	0.066	250
	BED-V205(%)-TL	1.8	6.2 (1.3-30)	5.2	0.0233	0.76	0.066	80
	BED-V400(%)-TL	2.7	15 (1.8-120)	6.2	0.0126	0.78	0.058	46
	BED-MAX-NPL-Gy	2.4	11 (1.3-92)	4.7	0.0301	0.72	0.087	540
	BED-Mean-NPL-Gy	2.4	11 (1.3-92)	4.7	0.0301	0.72	0.087	100
	BED-V120(%)-NPL	2.5	12 (1.4-98)	5.2	0.0228	0.73	0.09	22
	BED-V205(%)-NPL	2.4	11 (1.3-92)	4.7	0.0301	0.72	0.087	10
	BED-V400(ml)-NPL	2.4	11 (1.3-92)	4.7	0.0301	0.72	0.087	4.6
	BED-V400(%)-NPL	2.4	11 (1.3-92)	4.7	0.0301	0.72	0.087	1.1
	BED-MAX-WNL-Gy	2.4	11 (1.3-92)	4.7	0.0301	0.72	0.087	540
	BED-V400(ml)-WNL	2.4	11 (1.3-92)	4.7	0.0301	0.72	0.087	6.4
	BED-V400(%)-WNL	2.4	11 (1.3-92)	4.7	0.0301	0.72	0.087	0.38

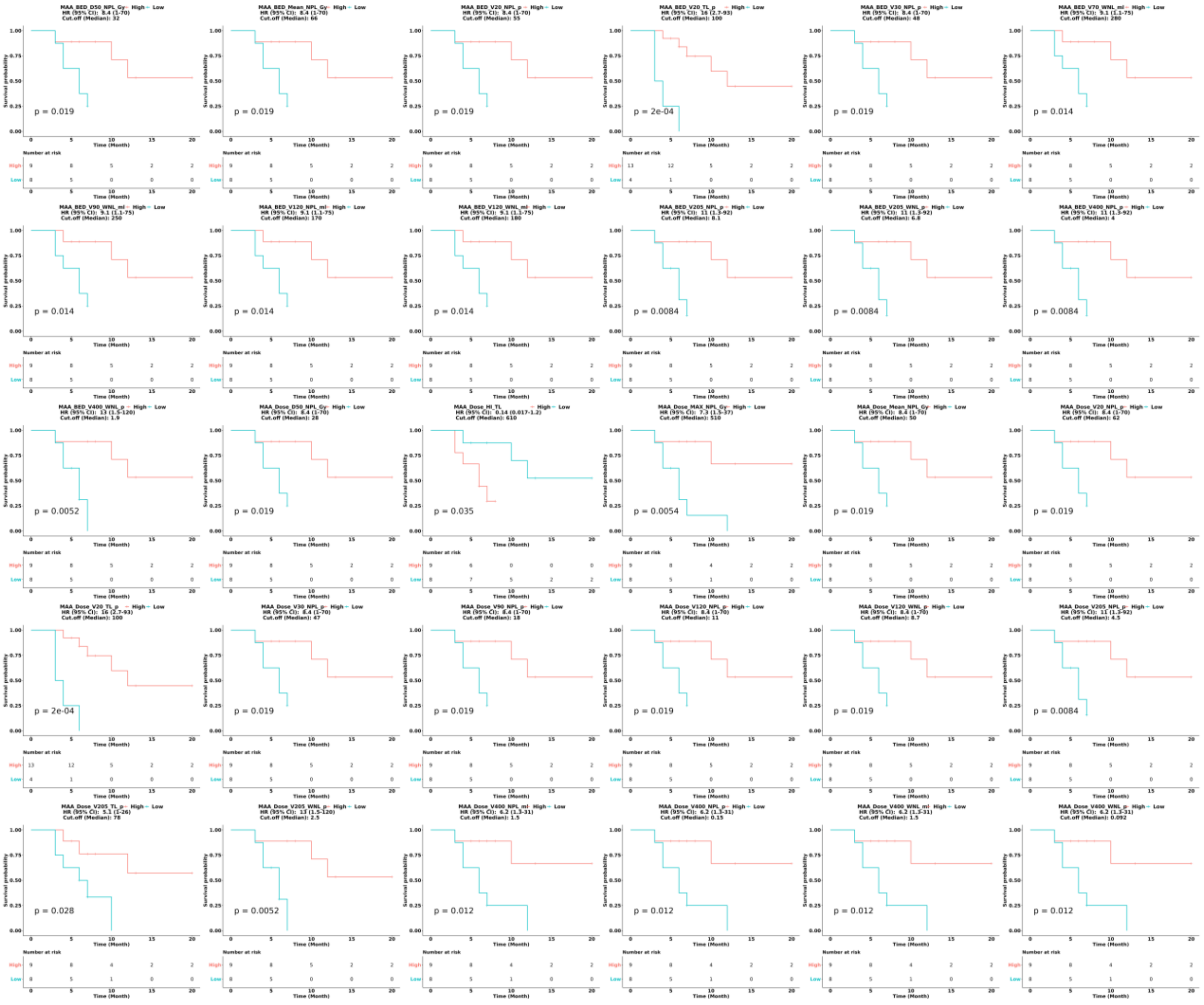

Figure 1. The Kaplan-Meier curves for dosimetry features from MAA found to be correlated with “PFS” during the univariate analysis.

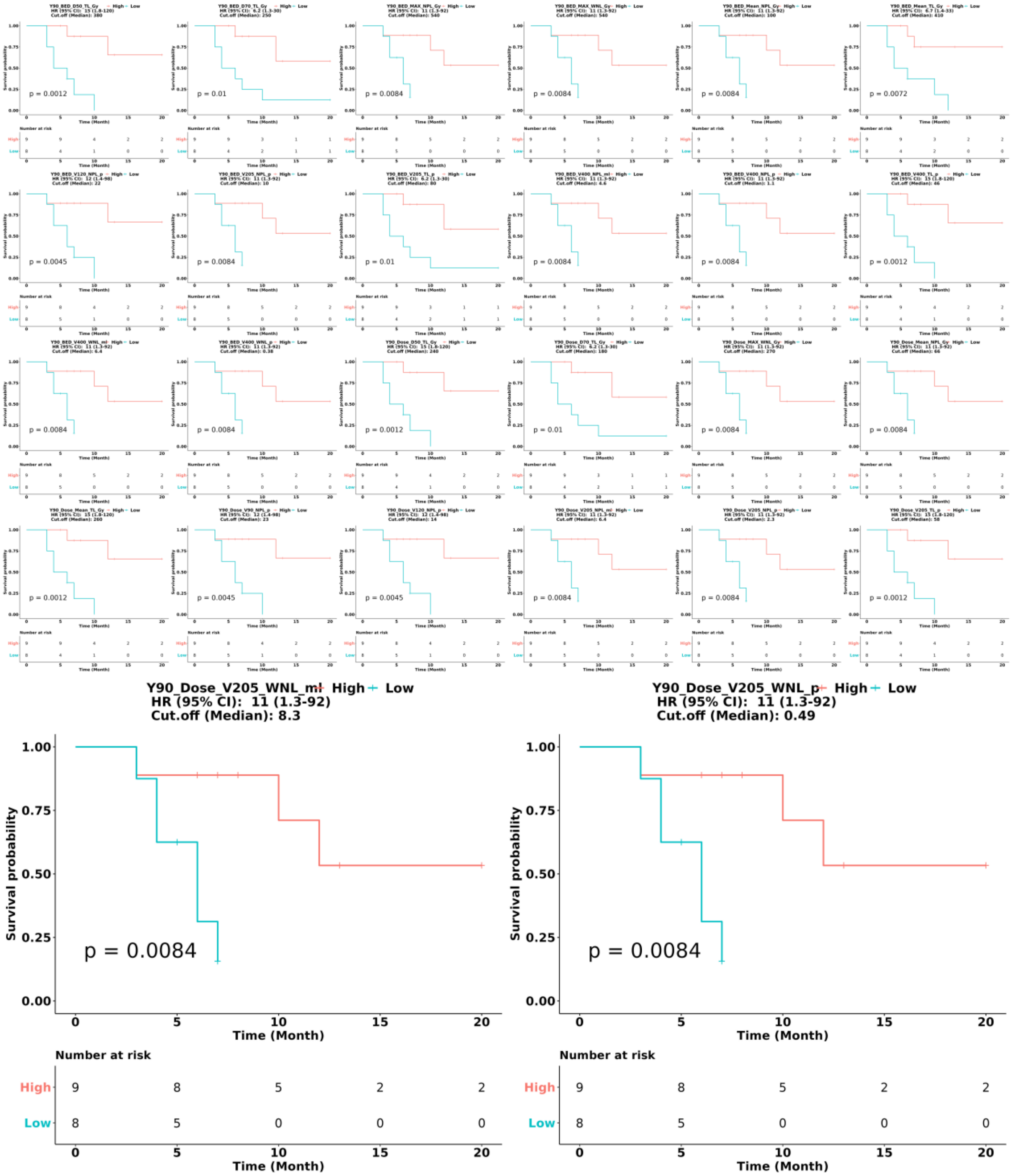

Figure 2. The Kaplan-Meier curves for Dosimetry features from ^{90}Y were found to be correlated with "PFS" during the univariate analysis.

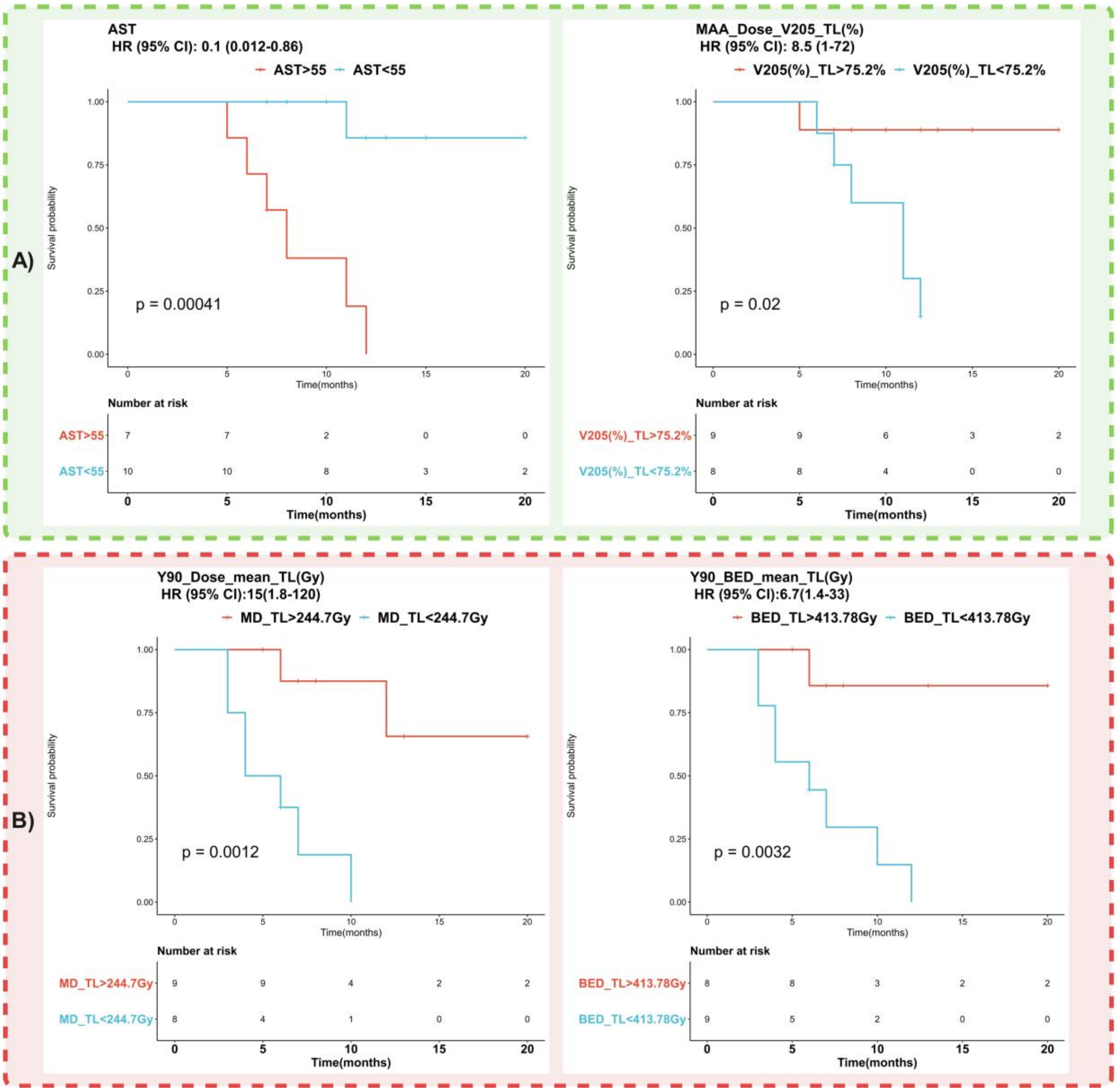

Figure 3. T illustrates the optimal cut-off values stratifying the Kaplan-Meier curves. In Panel A, statistically significant predictors of OS, including Aspartate Aminotransferase (AST) and MAA-Dose-V205_TL (%), exhibited optimum values of 55 (U/L) and 75.2%, respectively. Panel B showcases statistically significant predictors of PFS, such as Y90-Dose-mean-TL (Gy) and Y90-BED-mean-TL (Gy), with optimum values of 244.7 and 413.78 Gy, respectively.

Feature selection and Machine learning

Feature Selection methods:

1) Univariate concordance index(UCI)

UCI is a hybrid (filter and wrapper) feature selection method that works based on Cox proportional hazard regression. Spearman's rank correlation coefficient (rho) was used to omit the redundant features (rho>0.9). The remaining features were fed into a univariate Cox proportional hazard model. Bootstrap aggregation (100 repetitions) was performed on the training dataset, and Concordance Index (C-Index) was calculated. The top up-to-7 features with the highest C-index were selected.

Random survival Forest based feature selection methods

three wrapper feature selection methods working based on the Random survival Forest (RSF) algorithm, which consists of multiple decision trees, and each tree has a depth within the forest(1, 2):

2) minimum depth (MD)

The feature importance is calculated based on their depth in a tree (closeness to the root node). The root node has a depth of 0; its child node has a depth of 1, and so on; features that are closer to the root tree (or have the minimum depth) averaged across the forest are more important. The features were ranked based on their average depth across all trees.

3,4) Variable hunting (VH), Variable hunting with variable importance (VH.VIMP)

The feature selection started with splitting the dataset into train and test sets randomly and training an RSF model. Then, random features were selected by M-threshold. An initial model was formed by feeding the selected features, then features were added to the model until the joint variable importance is stabilized. The process was repeated 50 times and up to 7 closest features to the root (with minimal depths) were selected. The only difference between VH.VIMP and VH is that VH.VIMP uses the variable importance instead of minimal depth to rank the features.

5) Mutual Information (MI)

Mutual information (MI) operates by measuring the interdependence, shared information, or correlation between two variables. This methodology exhibits parallelized efficiency. MI calculation between two columns relies on a linear approximation established through correlation. The correlation between variables was identified using either Pearson's or Spearman's estimators. Additionally, the correlation within the data was computed using Somers' Dxy index.(3).

Machine learning models:

1) Cox Proportional Hazard regression (CoxPH):

In this model, the hazard function is calculated through the Cox Proportional hazards (Coxph) regression model which employes a subsequent equation as follows:

$$h(t) = h_0(t). \exp(\beta_1 x_1 + \beta_1 x_2 + \cdots + \beta_p x_p)$$

where, $h(t)$ represents the hazard function at time t, $h_0(t)$ is the baseline hazard function and β_1, β_2,…, β_p are the regression coefficients associated with covariates x_1, x_2,…, x_p. This formula enables us to evaluate the risk of any event at any given time, taking into account the impact of predators(4).

2) Generalized Linear Model Network (GLMN):

This network utilizes multiple interacting Generalized linear models (GLMs) which are statistical models that extend the linear regression model to handle non-normally distributed variables (e.g. Poisson, binormal, …). Each node of the GLMN represents a GLM and the connections between nodes represent the interactions(5).

3) GLM Boosting (GLMB):

This model combines the GLMs (described earlier) and boosting algorithms which are ensemble learning techniques. This technique builds sequentially weak learners (models with slightly better performance than random chance) to correct the mistakes and fit the residuals (the differences between the observed and predicted values) in an iterative way. In GLMB, GLMs serve as weak learners. These models are particularly useful when dealing with non-linear patterns in dataset or non-normally distributed response variables(6).

4) Random Survival Forest (RSF):

This model is an extension of Random Forest algorithm as an ensemble learning method, which works by building multiple decision trees and combining their predictions to increase accuracy and robustness. Each tree in the RSF is constructed using a random subset of data and a random subset of predictors. RSF naturally provides variable

importance of each predictor, identifying the most effective contributors in survival modeling. RSF is suitable for handling censored data and is a versatile tool for time-to-event analysis(1).

5) Survival Tree (ST)

Basically, this model is a decision tree and a statistical approach. These models can handle not only the time-to-event data but also censoring. Like the decision trees, a recursive partitioning process is used(7).

The Multiple Linear Regression (MLR) package 2.18 in R was utilized in this study. The hyperparameter tuning settings and packages that were used are summarized in supplemental-table 5.

Table 5. The models, R packages, and the hyperparameter settings used in this study.

Model	R package	Hyperparameters: range
**Coxph**	Survival	-
**RSF**	Random Forest SRC	ntree: 100, 500, 1000<br>mtry:1-10<br>node size: 1:20<br>splitrule: log-rank, logrankscore
**GLMB**	mboost	mstop: 50-500
**GLMN**	glmnet	S: 0.001-0.1<br>alpha: 0-1
**ST**	rpat	minsplit: 1-20<br>maxdepth: 1-30

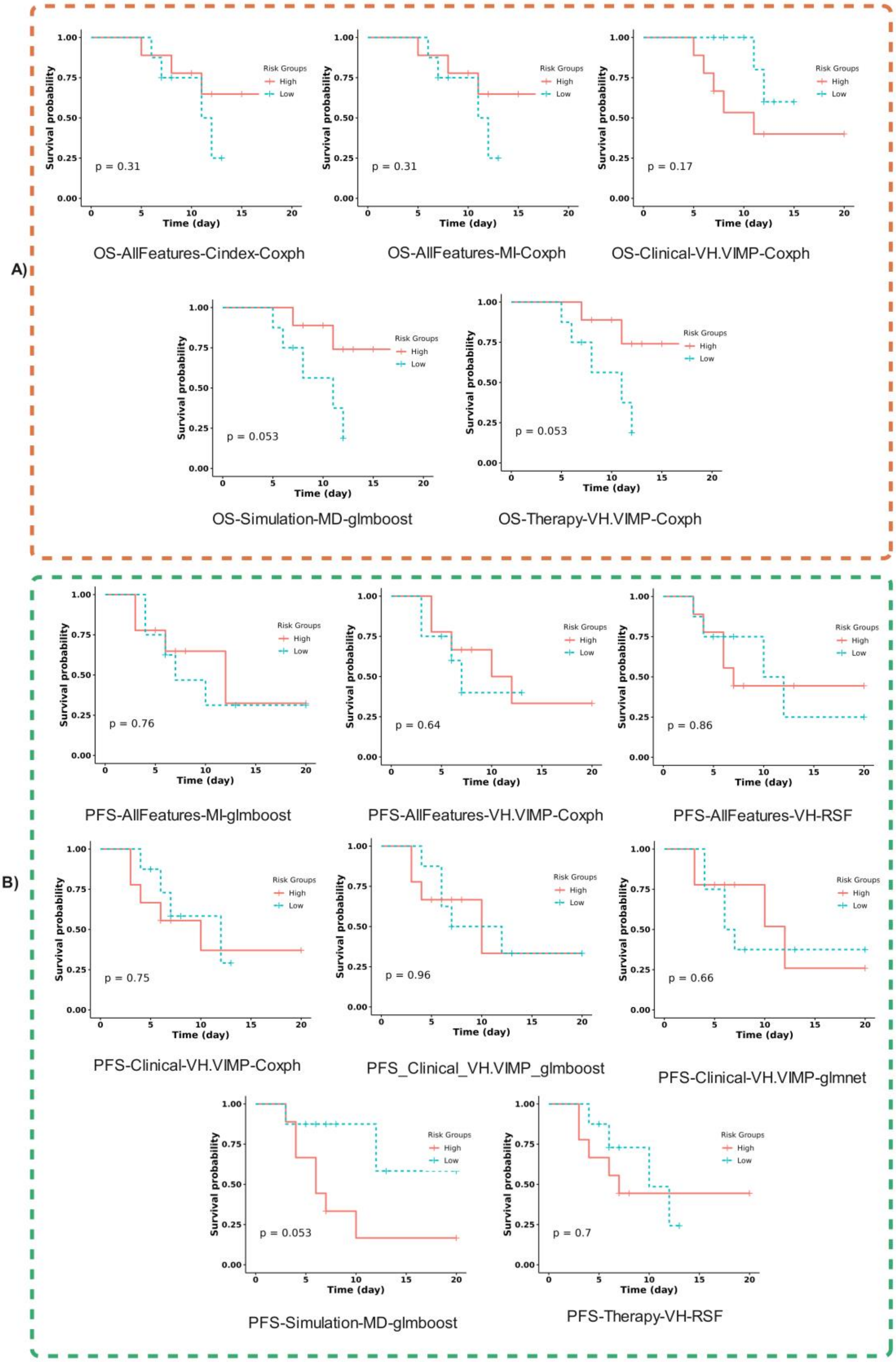

Figure 4. Kaplan-Meier curves for the high -performance prognostic models in terms of C-Index in each strategy predicting A) OS and B) PFS.

Table 6. The features selected in each fold through each feature selection methods applying on **All features** strategy to predict the **Overall survival.**

FS	*Fold 1*	*Fold 2*	*Fold 3*
Univariate C-index	Aim-of-RE<br>Hepatitis<br>ALT<br>AST<br>MAA-Dose-V30(ml)-WNL<br>LSF<br>ECOG	Y90-BED-HI-TL<br>Aim-of-RE<br>Hepatitis<br>Y90-Dose-V400(%)-WNL<br>Y90-BED-V30(%)-TL<br>Cirrhosis<br>WBC	Aim-of-RE<br>ECOG<br>Hepatitis<br>AST<br>MAA-BED-V20-TL-p<br>Y90-BED-V30-TL-p<br>MAA-BED-V30-TL-p
MI	Aim-of-RE<br>Hepatitis<br>ALT<br>AST<br>MAA-Dose-V30(ml)-WNL<br>LSF<br>ECOG	Y90-BED-HI-TL<br>Aim-of-RE<br>Hepatitis<br>Y90-Dose-V400(%)-WNL<br>Y90-BED-V30(%)-TL<br>Cirrhosis<br>WBC	Aim-of-RE<br>ECOG<br>Hepatitis<br>AST<br>MAA-BED-V20(%)-TL<br>Y90-BED-V30(%)-TL<br>MAA-BED-V30(%)-TL
VH	LSF<br>AST<br>ALT	MAA-Dose-MAX-TL-Gy<br>Y90-TNR-NPL<br>WBC	MAA-BED-MAX-TL-Gy<br>WBC<br>MAA-BED-V50(ml)-NPL<br>Y90-BED-V50(ml)-TL<br>Age
VH.VIMP	MAA-BED-V20(ml)-NPL<br>PVT<br>AST	Volume-WNL-ml<br>MAA-HI-BED-TL<br>Y90-Dose-V30(ml)-NPL	MAA-Dose-V400(%)-WNL<br>Y90-BED-V50(ml)-TL<br>AST
MD	Ascites<br>Platelet<br>MAA-TNR-WNL<br>ALT<br>AST<br>LSF<br>MAA-BED-V50(ml)-NPL	Platelet<br>Y90-BED-HI-TL<br>Y90-BED-MAX-TL-Gy<br>Y90-Dose-V400(ml)-TL<br>Y90-BED-Mean-TL-Gy<br>Y90-BED-V120(%)-NPL<br>MAA-BED-Min-WNL-Gy	AST<br>Y90-TNR-WNL<br>WBC<br>MAA-BED-V50(ml)-NPL<br>Platelet<br>Ascites<br>Y90-BED-V50(ml)-TL

Table 7. The features selected in each fold through each feature selection methods applying on **Clinical strategy** to predict the **Overall survival.**

FS	*Fold 1*	*Fold 2*	*Fold 3*
Univariate C-index	Aim-of-RE	Aim-of-RE	Aim-of-RE
	Hepatitis	Hepatitis	ECOG
	ALT	Cirrhosis	Hepatitis
	AST	WBC	AST
	ECOG	PVT	WBC
	Platelet	Bilirubin	ALT
	Age	AST	Age
MI	Aim-of-RE	Aim-of-RE	Aim-of-RE
	Hepatitis	Hepatitis	ECOG
	ALT	Cirrhosis	Hepatitis
	AST	WBC	AST
	ECOG	PVT	WBC
	Platelet	Bilirubin	ALT
	Age	AST	Age
VH	Age	Age	Bilirubin
	Bilirubin	WBC	AST
			Age
VH. VIMP	AST	Age	Ascites
	Platelet	Hepatitis	AST
			ALT
MD	Ascites	Platelet	AST
	Platelet	ALT	Platelet
	AST	WBC	WBC
	ALT	Cirrhosis	Age
	Bilirubin	AST	Albumin
	AFP	AFP	Ascites
	Albumin		Hb

Table 8. The features selected in each fold through each feature selection methods applying on **Simulation strategy** to predict the **Overall survival**.

FS	*Fold 1*	*Fold 2*	*Fold 3*
Univariate C-index	Dose-V30(ml)-WNL<br>LSF<br>BED-V20(ml)-NPL<br>BED-V50(ml)-NPL<br>TNR-WNL<br>BED-Volume(ml)-NPL<br>BED-Volume(ml)-WNL	Dose-HI-TL<br>BED-HI-TL<br>Dose-V20(%)-TL<br>BED-V20(%)-TL<br>Dose-D95-WNL-Gy<br>Dose-D98-WNL-Gy<br>Dose-V205(%)-TL	BED-V20(%)-TL<br>BED-V30(%)-TL<br>Dose-V205(ml)-TL<br>BED-V205(ml)-TL<br>Dose-V400(%)-WNL<br>TNR-NPL<br>TNR-WNL
MI	Dose-V30(mL)-WNL<br>LSF<br>BED-V20(ml)-NPL<br>BED-V50(ml)-NPL<br>TNR-WNL<br>BED-Volume(ml)-NPL<br>BED-Volume(ml)-WNL	Dose-HI-TL<br>BED-HI-TL<br>Dose-V20(%)-TL<br>BED-V20(%)-TL<br>Dose-D95-WNL-Gy<br>Dose-D98-WNL-Gy<br>Dose-V205(%)-TL	BED-V20(%)-TL<br>BED-V30(%)-TL<br>Dose-V205(ml)-TL<br>BED-V205(ml)-TL<br>Dose-V400(%)-WNL<br>TNR-NPL<br>TNR-WNL
VH	Dose-Min-NPL-Gy<br>BED-V50(ml)-NPL<br>TNR-WNL	BED-MAX-TL-Gy<br>BED-Min-WNL-Gy	BED-D50-TL-Gy<br>Dose-V205(ml)-TL
***VH.* VIMP**	Dose-Volume(ml)-NPL<br>Dose-Min-NPL-Gy<br>Dose-V30(ml)-WNL	Dose-HI-TL<br>Dose-V205-TL-p<br>BED-HI-TL	Dose-V400(%)-WNL<br>BED-D50-TL-Gy<br>TNR-WNL
MD	LSF<br>BED-D95-NPL-Gy<br>BED-V50(ml)-NPL<br>TNR-WNL<br>Dose-Min-NPL-Gy<br>BED-V20(%)-NPL<br>BED-V90(ml)-NPL	BED-Min-WNL-Gy<br>BED-Mean-TL-Gy<br>BED-MAX-TL-Gy<br>Dose-MAX-TL-Gy<br>Dose-V205(ml)-TL<br>Dose-Min-NPL-Gy<br>BED-V400(ml)-TL	BED-D50-TL-Gy<br>Dose-V205(ml)-TL<br>BED-V205(ml)-TL<br>Dose-Min-NPL-Gy<br>BED-V50(ml)-NPL<br>BED-MAX-WNL-Gy<br>TNR-WNL

Table 9. The features selected in each fold through each feature selection method applying on **Therapy strategy** to predict the **Overall survival**.

FS	*Fold 1*	*Fold 2*	*Fold 3*
Univariate C-index	Dose-V30(%) -TL<br>Dose-Min-NPL-Gy<br>Dose-V400(ml)-TL<br>Dose-V400(%)-WNL<br>BED-Volume(ml)-WNL<br>BED-D50-TL-Gy<br>BED-V400(%)-NPL	BED-HI-TL<br>BED-MAX-TL-Gy<br>Dose-V400(ml)-TL<br>BED-V120(ml)-WNL<br>Dose-V30(ml)-NPL<br>BED-D50-NPL-Gy<br>Dose-MAX-NPL-Gy	BED-V50(ml)-TL<br>Dose-Min-NPL-Gy<br>TNR-NPL<br>BED-V205(ml)-TL<br>Dose-V205(ml)-TL<br>TNR-WNL<br>BED-V400(ml)-TL
MI	Dose-V30(%) -TL<br>Dose-Min-NPL-Gy<br>Dose-V400(ml)-TL<br>Dose-V400(%)-WNL<br>BED-Volume(ml)-WNL<br>BED-D50-TL-Gy<br>BED-V400(%)-NPL	BED-HI-TL<br>Dose-V400(%)-WNL<br>BED-V30(%)-TL<br>BED-MAX-TL-Gy<br>Dose-V400(ml)-TL<br>Dose-MAX-NPL-Gy<br>Dose-V205(ml)-NPL	BED-V30(%)-TL<br>BED-V50(ml)-TL<br>Dose-Min-NPL-Gy<br>TNR-NPL<br>BED-V205(ml)-TL<br>TNR-WNL<br>BED-V50(%)-TL
VH	Dose-Min-NPL-Gy<br>BED-V120(%) -WNL	Dose-V400(ml) -TL<br>BED-V120(%)-NPL<br>Dose-MAX-NPL-Gy	BED-V50(ml)-TL<br>Dose-Min-NPL-Gy
VH. VIMP	Dose-Min-NPL-Gy<br>Dose-V400(%)-TL<br>BED-MAX-TL-Gy	Dose-V30(ml)-NPL<br>BED-D50-NPL-Gy<br>BED-HI-TL	Dose-V50(%)-TL<br>BED-V50(ml)-TL
MD		BED-HI-TL<br>BED-MAX-TL-Gy<br>TNR-NPL<br>BED-Mean-TL-Gy<br>TNR-WNL<br>Dose-V400(%)-TL<br>BED-V120(%)-NPL	Dose-MAX-NPL-Gy<br>Dose-Min-NPL-Gy

Table 10. The features selected in each fold through each feature selection methods applying on **All features** strategy to predict the **progression free survival.**

FS	*Fold 1*	*Fold 2*	*Fold 3*
Univariate C-index	Ascites	Hepatitis	MAA-BED-V30(%)-TL
	MAA-Dose-V20(%)-TL	MAA-Dose-V20(%)-TL	Cirrhosis
	MAA-BED-V20(%)-TL	MAA-BED-V20(%)-TL	Y90-Dose-V400(%)-WNL
	Y90-BED-V30(%)-TL	Y90-TNR-WNL	Previous-EBRT-or-other-treatments
	Hepatitis	Aim-of-RE	Hepatitis
	MAA-Dose-V30(%)-TL	Ascites	MAA-Dose-V400(%) -NPL
	MAA-BED-V30(%)-TL	Cirrhoses	MAA-BED-V50(%) -TL
MI	Ascites	Hepatitis	MAA-BED-V30(%)-TL
	MAA-Dose-V20(%)-TL	MAA-Dose-V20(%)-TL	Cirrhosis
	MAA-BED-V20(%)-TL	MAA-BED-V20(%)-TL	Y90-Dose-V400(%)-WNL
	Y90-BED-V30(%)-TL	Y90-TNR-WNL	Previous-EBRT-or-other-treatments
	Hepatitis	Aim-of-RE	Hepatitis
	MAA-Dose-V30(%)-TL	Ascites	MAA-Dose-V400(%) -NPL
	MAA-BED-V30(%)-TL	Cirrhosis	MAA-BED-V50(%) -TL
VH	MAA-BED-V90(ml)-NPL	MAA-BED-V400(ml)-NPL	Y90-BED-V205(%)-WNL
	MAA-TNR-WNL	MAA-Dose-V205(%)-TL	MAA-Dose-V400(%)-NPL
	MAA-Dose-V20(%)-WNL	MAA-TNR-WNL	MAA-BED-V400(%)-NPL
	MAA-BED-V50(ml)-NPL	Y90-Dose-V400(ml)-TL	Y90-TNR-WNL
	LSF	MAA-Dose-MAX-WNL-Gy	AST
	MAA-BED-V205(ml)-TL	MAA-BED-V20(%)-TL	MAA-Dose-MAX-WNL-Gy
		MAA-Dose-V205(%)-NPL	Y90-BED-Min-TL-Gy
VH. VIMP	MAA-BED-V50(ml)-NPL	MAA-Dose-MAX-WNL-Gy	MAA-Dose-V205(%) -NPL
	MAA-BED-V90(ml)-NPL	MAA-Dose-V20(%)-TL	MAA-Dose-V400(%) -NPL
	Y90-Dose-V30(ml)-WNL	MAA-Dose-V205(%)-TL	Y90-Dose-V205(%) -NPL
	Y90-BED-Mean-TL-Gy	Y90-TNR-WNL	Y90-BED-V205(ml)-NPL
	Y90-BED-V400(%)-TL		Platelet
MD	Volume-NPL-ml	Y90-TNR-WNL	MAA-Dose-MAX-WNL-Gy
	Age	MAA-Dose-MAX-WNL-Gy	Volume-NPL-ml
	MAA-Dose-V20(%)-NPL	MAA-Dose-V400(%) -NPL	
	MAA-BED-V90(ml)-NPL	MAA-Dose-V205(%) -TL	
	MAA-BED-V50(ml)-NPL	ALT	
	Volume-WNL-ml	MAA-BED-V20(ml)-WNL	
		Y90-BED-V400(%)-TL	

Table 11. The features selected in each fold through each feature selection methods applying on **Clinical strategy** to predict the **progression free survival**.

FS	*Fold 1*	*Fold 2*	*Fold 3*
Univariate C-index	Ascites<br>Hepatitis<br>Age<br>Cirrhosis<br>Aim-of-RE<br>Previous-EBRT-or-other-treatments<br>WBC	Hepatitis<br>Aim-of-RE<br>Ascites<br>Cirrhosis<br>Previous-EBRT-or-other-treatments<br>PVT<br>AST	Cirrhosis<br>Previous-EBRT-or-other-treatme<br>Hepatitis<br>Aim-of-RE<br>Ascites<br>Platelet<br>ECOG
MI	Ascites<br>Hepatitis<br>Age<br>Cirrhosis<br>Aim-of-RE<br>Previous-EBRT-or-other-treatments<br>WBC	Hepatitis<br>Aim-of-RE<br>Ascites<br>Cirrhosis<br>Previous-EBRT-or-other-treatments<br>PVT<br>AST	Cirrhosis<br>previous-EBRT-or-other-treatments<br>Hepatitis<br>Aim-of-RE<br>Ascites<br>Platelet<br>ECOG
VH	Hb<br>Age	Bilirubin<br>ALT	Age<br>Aim-of-RE
VH. VIMP	Ascites<br>AST	AST<br>ECOG	Age<br>Previous-EBRT-or-other-treatments
MD	Age<br>Albumin	AST<br>Age<br>ALT<br>AFP<br>Platelet<br>Albumin	Platelet<br>Cirrhosis<br>Age<br>Bilirubin<br>AFP<br>AST

Table 12. The features selected in each fold through each feature selection methods applying on **Simulation strategy** to predict the **progression free survival**.

FS	*Fold 1*	*Fold 2*	*Fold 3*
Univariate C-index	BED-V90(ml)-NPL	Dose-V20(%)-TL	BED-Min-WNL-Gy
	Dose-V20(%)-WNL	BED-V400(%)-TL	Dose-V400(%)-NPL
	Dose-V50(%)-TL	BED-V20(%)-TL	BED-V205(%)-NPL
	BED-V50(ml)-NPL	Dose-MAX-WNL-Gy	BED-V70(%)-TL
	Volume(ml)_NPL	Dose-V400(%)-NPL	BED-V50(%)-TL
	Dose-V30(%) -NPL	Dose-V205(%)-TL	Dose-V205(%)-WNL
	Dose-V30(%)-TL	BED-V50(%)-TL	Dose-MAX-WNL-Gy
MI	Dose-V20(%)-TL	Dose-V20(%)-TL	BED-V30(%)-TL
	BED-V20(%)-TL	BED-V20(%)-TL	Dose-V400(%)-NPL
	Dose-V30(%)-TL	BED-V30(%)-TL	BED-V50(%)-TL
	BED-V30(%)-TL	Dose-V400(%)-NPL	Dose-V50(%)-TL
	Dose-V50(%)-TL	Dose-MAX-WNL-Gy	BED-Min-WNL-Gy
	Dose-V20(%)-WNL	BED-V50(%)-TL	BED-V70(%)-TL
	BED-V50(ml)-NPL	BED-V400(%)-TL	Dose-MAX-WNL-Gy
VH	LSF	Dose-V205(%)-TL	BED-V400(ml)-NPL
	Dose-Volume-(ml)-NPL	TNR-WNL	Dose-D98-WNL-Gy
	BED-V90-(ml)-NPL	BED-V400(%)-TL	TNR-WNL
	TNR-NPL	Dose-Mean-TL-Gy	Dose-MAX-WNL-Gy
VH. VIMP	Dose-MAX-WNL-Gy	Dose-V20(%)-TL	Dose-V50(%)-TL
	Dose-V30- (%)-TL	Dose-V205(%)-TL	Dose-V205(%)-WNL
	Dose-V50- (%)-TL	BED-V20(%)-TL	BED-V70(%)-TL
	BED-V90(ml)-NPL	BED-V30(%)-TL	BED-V400(ml)-NPL
MD	Volume(ml)-NPL	Dose-V205(%)-TL	Dose-MAX-WNL-Gy
	Dose-MAX-WNL-Gy	Dose-MAX-WNL-Gy	Dose-MAX-TL-Gy
		TNR-WNL	Dose-D70-TL-Gy
		BED-V30(%)-TL	Dose-Min-NPL-Gy
		Dose-V205(ml)-TL	
		BED-V20(%)-TL	
		Dose-V400(ml)-TL	

Table 13. The features selected in each fold through each feature selection method applying on **Therapy strategy** to predict the **progression free survival**.

FS	*Fold 1*	*Fold 2*	*Fold 3*
Univariate C-index	BED-V30(%)-TL	TNR-WNL	Dose-V400(%)-WNL
	BED-Min-TL-Gy	Dose-V400(%)-TL	Dose-MAX-NPL-Gy
	BED-V400(%)-TL	Dose-V400(%-WNL	Dose-V205(%)-NPL
	BED-Mean-TL-Gy	BED-D50-TL-Gy	BED-V205(%)-NPL
	BED-V50(%)-TL	BED-V400(%)-TL	BED-MAX-WNL-Gy
	Dose-V50(%)-TL	Dose-V30(%)-TL	BED-V205(ml)-WNL
	Dose-V120(%)-TL	BED-V205(ml)-TL	BED-V205(%)-WNL
MI	BED-V30(%)-TL	TNR-WNL	Dose-V400(%)-WNL
	BED-Min-TL-Gy	Dose-V400(%)-TL	Dose-MAX-NPL-Gy
	BED-V400(%)-TL	Dose-V400(%)-WNL	Dose-V205(%)-NPL
	BED-Mean-TL-Gy	BED-D50-TL-Gy	BED-V205(%)-NPL
	BED-V50(%)-TL	BED-V400(%)-TL	BED-MAX-WNL-Gy
	Dose-V50(%)-TL	Dose-V30(%)-TL	BED-V205(ml)-WNL
	Dose-V120(%)-TL	BED-V205(ml)-TL	BED-V205(%)-WNL
VH	Dose-V120(%)-TL	BED-V120(ml)-WNL	Dose-V205(%)-TL
	BED-Min-TL-Gy	BED-V205(%)-NPL	Dose-MAX-NPL-Gy
	BED-V20(ml)-WNL	Dose-V205(%) -NPL	BED-V205(%)-NPL
	Dose-V50(%)-TL		BED-V205(%)-WNL
VH. VIMP	Dose-V120(%)-TL	Dose-V400(%)-TL	Dose-MAX-NPL-Gy
	Dose-V400(ml)-TL	BED-D50-TL-Gy	BED-MAX-WNL-Gy
	BED-Min-TL-Gy	TNR-WNL	BED-V120(ml)-NPL
			BED-V205(%)-NPL
MD	BED-V400(%)-NPL	TNR-WNL	Dose-V205(%)-NPL
	BED-V20(ml)-WNL	BED-V400(%)-TL	Dose-MAX-NPL-Gy
	BED-V120(ml)-TL	Dose-V400(%)-TL	BED-V205(%)-NPL
	BED-V400(ml)-WNL	BED-D50-TL-Gy	BED-V205(%)-WNL
	BED-Min-TL-Gy	BED-V205(ml)-TL	BED-V205(ml)-WNL
	Dose-Min-NPL-Gy	Dose-Mean-WNL-Gy	TNR-WNL
	BED-V30(ml)-NPL	BED-V400(ml)-TL	BED-V120(ml)-NPL

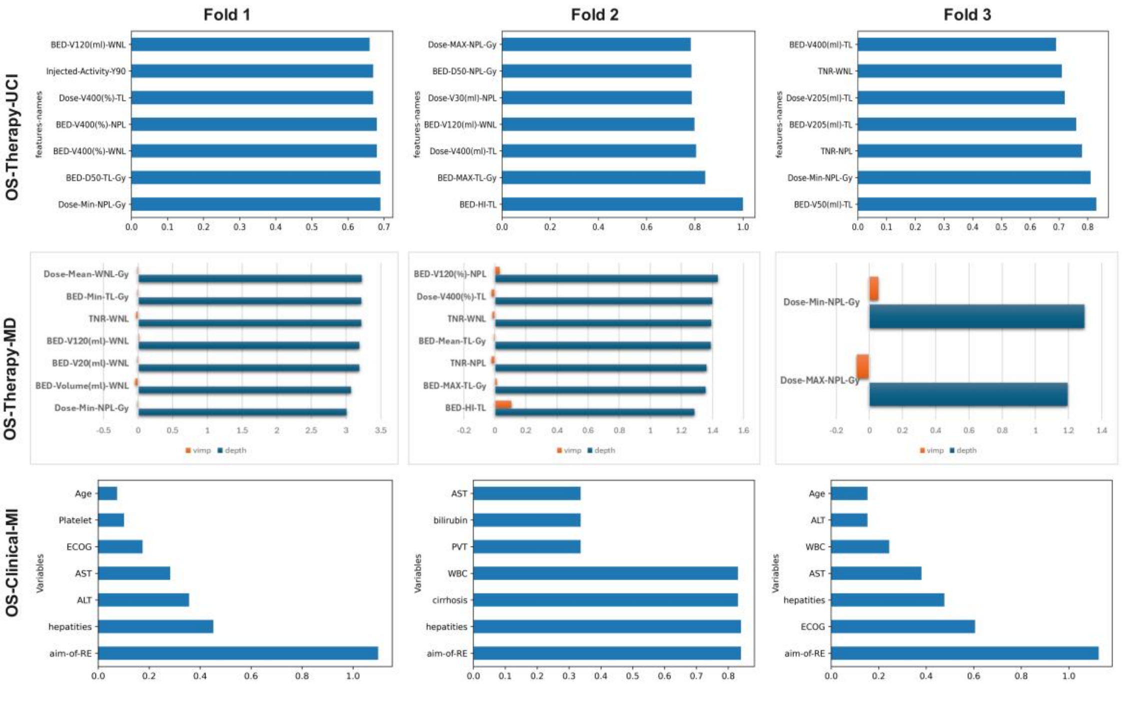

Figure 5. illustration of the selected features and their significance in constructing the overall survival models for selected and well-performed models.

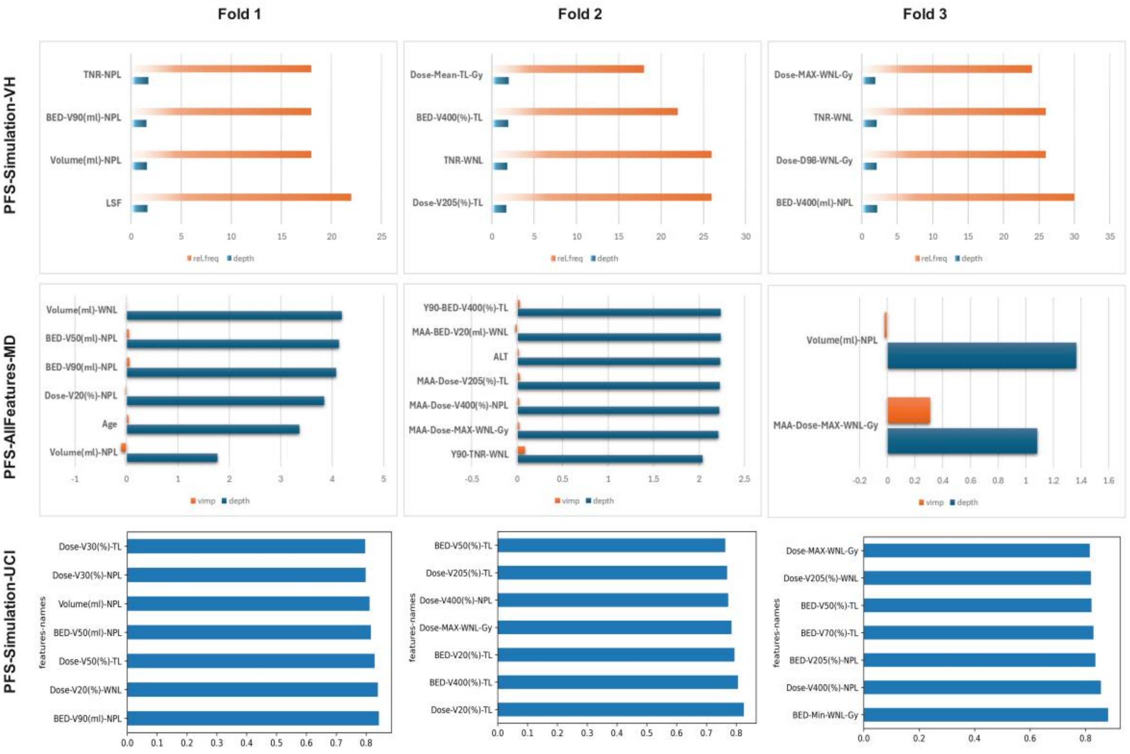

Figure 6. Selected features and their importance, contributing to building the selected and well-performed progression free survival models.

References

1. Ishwaran H, Kogalur UB, Blackstone EH, Lauer MS. Random survival forests. 2008.
2. Ishwaran H, Kogalur UB, Gorodeski EZ, Minn AJ, Lauer MS. High-dimensional variable selection for survival data. Journal of the American Statistical Association. 2010;105(489):205-17.
3. De Jay N, Papillon-Cavanagh S, Olsen C, El-Hachem N, Bontempi G, Haibe-Kains B. mRMRe: an R package for parallelized mRMR ensemble feature selection. Bioinformatics. 2013;29(18):2365-8.
4. Andersen PK, Gill RD. Cox's regression model for counting processes: a large sample study. The annals of statistics. 1982:1100-20.
5. Simon N, Friedman J, Hastie T, Tibshirani R. Regularization paths for Cox's proportional hazards model via coordinate descent. Journal of statistical software. 2011;39(5):1.
6. Hofner B, Mayr A, Robinzonov N, Schmid M. Model-based boosting in R: a hands-on tutorial using the R package mboost. Computational statistics. 2014;29:3-35.
7. Breiman L. Classification and regression trees: Routledge; 2017.
